# Integrative analysis of transcriptomics, single-cell RNA sequencing, and GraphBAN identifies *de novo* lipogenesis-associated genes and their potential roles in diabetic retinopathy

**DOI:** 10.3389/fimmu.2026.1803639

**Published:** 2026-04-07

**Authors:** Zhenghong Jin, Yichen Dong, Rong Xue, Xialian Fan, Guangming Wan

**Affiliations:** 1 Department of Ophthalmology, The First Affiliated Hospital of Zhengzhou University, Zhengzhou, Henan, China; 2 Engineering Research Center of Fundus Disease and Ocular Trauma Prevention and Treatment, Zhengzhou, Henan, China; 3 International Joint Research Laboratory for Ocular Immunology and Retinal Injury Repair, Zhengzhou, Henan, China

**Keywords:** *de novo* lipogenesis, diabetic microvascular complications, diabetic retinopathy, GraphBAN model, molecular dynamics simulation, single-cell RNA sequencing

## Abstract

**Background:**

Diabetic retinopathy (DR) represents a key microvascular disorder resulting from prolonged diabetes mellitus. Hyperglycemia, a common driver of diabetes-related vision loss, can pathologically activate *de novo* lipogenesis (DNL); however, the underlying mechanisms remain incompletely understood. This work sought to profile signature genes correlated with DNL and DR, and offer novel insights into the disease’s underlying pathogenesis.

**Methods:**

DR-related transcripts and DNL-associated genes were obtained from public databases. Genes with DNL-related signatures associated with DR were identified through integrated bioinformatics analyses. These genes were incorporated into a nomogram, and their functional characteristics were further analyzed. The GraphBAN model was applied to predict potential gene–compound interactions, evaluate binding affinity, and assess docking stability. Single-cell RNA sequencing data were used to characterize the cellular landscape and to map gene expression patterns to specific cell populations within DR fibrous membranes, providing cellular context for the transcriptomic findings. Finally, RT-qPCR was performed to measure whole-blood expression of key genes between DR patients and control subjects.

**Results:**

*AHR* and *SLC1A5* were identified as genes potentially associated with DR. RT-qPCR confirmed significant upregulation of both genes in the DR group. A nomogram established using these genes suggested that the predicted risk of DR increased with higher total scores. Functional enrichment analysis revealed that these genes were implicated in multiple biological pathways closely associated with DR pathogenesis. Integration of the GraphBAN model with compound screening predicted that two and fourteen compounds may interact with *AHR* and *SLC1A5*, respectively, and molecular docking suggested potential binding between these genes and several candidate compounds. At the single-cell level, conventional dendritic cell type 2 (cDC2) and classical monocytes showed notable transcriptional activity related to DR-associated pathways. cDC2 displayed relatively high activity across multiple metabolic pathways. During the differentiation of these cell populations, the selected genes exhibited dynamic expression patterns.

**Conclusion:**

This exploratory study identified two DNL-associated candidate genes and explored their potential roles in DR-related molecular processes. These preliminary findings offer novel insights into the pathogenesis of DR and lay a foundational basis for future investigations into DNL-associated pathways and the development of therapeutic strategies targeting DR.

## 1 Introduction

Diabetes mellitus (DM) encompasses a spectrum of persistent metabolic disorders marked by impaired glucose regulation, primarily caused by deficiencies in insulin production, cellular response to insulin, or a combination of both, resulting in prolonged elevated blood sugar levels. It represents a major global health challenge because of its association with multiple organ complications (1). Diabetic retinopathy (DR) emerges as a predominant microvascular consequence of DM, mediated by chronic hyperglycemia and oxidative stress. These pathological processes lead to damage of retinal blood vessels, retinal edema, and pathological neovascularization, which may ultimately result in vision loss or blindness (2). Currently, DR affects approximately 30–40% of individuals with diabetes, corresponding to more than 100 million people worldwide. Future projections anticipate a dramatic rise in DR cases, with estimates predicting the condition may affect as many as 161 million people by 2045 (3–6). From a clinical perspective, DR follows a characteristic progressive course, advancing from non-proliferative DR (NPDR), which is distinguished by retinal vascular abnormalities, to proliferative DR (PDR), a late stage hallmarked by pathological neovascularization. Diabetic macular edema (DME) can develop at any stage of DR and represents a leading cause of vision impairment in affected patients (7). In current clinical practice, the standard-of-care therapeutic strategies for DR are broadly categorized into three major classes: laser-based retinal coagulation, intraocular administration of VEGF inhibitors, and surgical vitreous removal for severe cases (8–10). However, the concept of “metabolic memory” suggests that retinal damage may continue to progress even after glycemic control is achieved (11). Thus, a deeper mechanistic understanding of DR is critical to developing optimized diagnostic and therapeutic approaches.

*De novo* lipogenesis (DNL) is a metabolic process that converts carbohydrates into fatty acids for lipid synthesis. Proper regulation of this pathway is essential for maintaining metabolic homeostasis (12), whereas dysregulation of DNL has been associated with insulin resistance (13, 14). In the context of DR, hyperglycemia-induced activation of the DNL pathway may contribute to lipotoxicity, retinal inflammation, vascular dysfunction, and neurodegeneration (15). Current research strongly supports the notion that DNL plays a fundamental role in the early development of DR. Previous studies have reported elevated DNL activity in diabetic retinas, particularly in photoreceptor cells, and have shown that reducing glucose-driven lipid synthesis in these cells can alleviate retinal pathology (16, 17). While these observations imply that abnormal lipid metabolism in retinal tissue may trigger DR onset, the exact underlying biological processes require additional investigation.

Single-cell RNA sequencing (scRNA-seq) is a state-of-the-art high-throughput sequencing platform that enables unbiased genome-wide transcriptomic profiling at single-cell resolution. This approach allows detailed characterization of cellular heterogeneity within complex tissues and facilitates the identification of distinct cell types, functional states, and gene expression patterns (18). Using this technology, previous studies have reported alterations in the proportions of retinal cell populations and transcriptional reprogramming of retinal Müller glial cells and microglia in diabetic retinopathy. These studies have also highlighted inflammatory, angiogenic, and metabolic signaling pathways that may be associated with disease development, providing valuable insights into the multicellular mechanisms involved in DR pathogenesis (19).

In this study, an integrated multi-omics approach was used to investigate the potential role of DNL in DR. Bulk RNA-seq data were subjected to comprehensive bioinformatics analysis to screen for DNL-associated differentially expressed genes (DEGs), followed by functional characterization of the identified genes. Machine learning-based analytical algorithms were next implemented to screen for candidate DNL-linked genes with clinical relevance to DR. In addition, scRNA-seq data were used to characterize the cellular landscape, examine intercellular communication patterns, and perform *pseudotim*e trajectory analysis to explore potential dynamic changes in gene expression across cell populations. Finally, the GraphBAN model was employed to predict potential interactions between candidate genes and compounds, and these interactions were further evaluated through molecular docking and molecular dynamics simulations. Together, these exploratory analyses aimed to provide novel insights into DNL-related molecular processes in DR, and to lay preliminary groundwork for future diagnostic biomarker and therapeutic target research.

## 2 Materials and methods

### 2.1 Data sources

Transcriptomic data related to human DR were obtained from the publicly accessible Gene Expression Omnibus (GEO) database (https://www.ncbi.nlm.nih.gov/geo/). The GSE221521 dataset (platform: GPL24676) included 69 leukocyte samples from patients with DR and 50 leukocyte samples from control subjects. The GSE102485 dataset (platform: GPL18573) contained 22 retinal neovascular proliferative membrane samples from patients with PDR and 3 normal retinal samples. The GSE94019 dataset (platform: GPL11154) comprised 9 fibrovascular membrane endothelial cell samples from DR patients and 4 control samples. In addition, the single-cell sequencing dataset GSE165784 (platform: GPL20795) included 5 fibrous membrane samples from patients with DR. Genes associated with DNL were extracted from the GeneCards database (https://www.genecards.org/). To improve specificity and include genes with stronger associations with DNL, a relevance score threshold greater than 25 was applied. This filtering process yielded a total of 575 DNL-related genes (**Supplementary Table 1**) (20).

### 2.2 Differential expression analysis

To identify genes showing notable transcriptional alterations associated with DR, differential expression analysis was conducted via the DESeq2 package (v1.40.2) (21). Two datasets were analyzed independently: GSE221521 (DR vs. Control), reflecting systemic changes in peripheral blood, and GSE102485 (PDR vs. Normal retina), representing local transcriptional alterations in advanced retinal pathology. The analysis utilized raw gene expression count data as direct input for DESeq2. The criteria for identifying DEGs were set as |log2 fold change (FC)| values exceeding 0.5 with p-values below 0.05. To eliminate potential tissue-specific bias and identify genes with consistent DR association, upregulated and downregulated genes identified from the two independent datasets were subjected to separate intersection analysis. This strategy aimed to identify genes showing consistent dysregulation in both peripheral blood and retinal tissue. The overlapping genes were intersected with the list of DNL-related genes to identify genes with DNL-associated expression signatures.

### 2.3 Functional enrichment and interaction network of directional genes

To explore the biological mechanisms underlying these candidate genes, enrichment analyses were conducted utilizing the clusterProfiler package (v4.15.0) (22). Gene Ontology (GO) and Kyoto Encyclopedia of Genes and Genomes (KEGG) analyses were performed with statistical significance defined as adjusted p-values below 0.05. Potential protein–protein interactions were analyzed via STRING (https://string-db.org/), setting an interaction score threshold greater than 0.4.

### 2.4 Machine learning

To further screen for feature genes correlated with diabetic retinopathy, this study employed the GSE221521 dataset and integrated four machine learning algorithms for feature selection, adopting an intersection strategy to enhance the robustness of the results. First, the least absolute shrinkage and selection operator (LASSO) regression was applied for sparse feature screening. This analysis was performed using the cv.glmnet function from the glmnet package (v4.1.8) (23), with parameters set to family = “binomial” (binary logistic regression) and alpha = 1 (LASSO penalty). Ten-fold cross-validation was conducted to select the optimal penalty parameter (lambda.min) based on the minimum cross-validated binomial deviance, and genes with non-zero regression coefficients in the optimal model were retained. Second, the Boruta algorithm was used to evaluate gene importance. This algorithm identifies truly predictive features by constructing a random forest model and comparing the importance scores of original features with those of randomly generated “shadow” features. The analysis was performed using the Boruta package (v8.0.0) (24), with the number of decision trees set to 500 (ntree = 500). The algorithm was run iteratively until all features were confirmed or rejected, and genes classified as “confirmed” were retained as important features. Third, the random forest algorithm was employed to rank feature importance. This was implemented using the randomForest package (v4.7.1.2) (25). The key parameter mtry (the number of variables randomly sampled as candidates at each split) was optimized using the tuneRF function. The optimization process was performed with a step factor of 1.5 (stepFactor = 1.5), a model performance improvement threshold of 0.05 (improve = 0.05), and an initial tree number of 100 (ntreeTry = 100). The final model was constructed using the optimal mtry value, with the total number of decision trees set to 500 (ntree = 500). The top five genes based on importance scores were extracted. Fourth, the XGBoost algorithm was applied for modeling and feature selection. This was implemented using the xgboost package (v2.1.1.1) (26). The model was configured for binary logistic classification (objective = “binary:logistic”) with the number of boosting rounds set to 25 (nrounds = 25). After model training, the xgb.importance function was used to calculate the contribution score for each feature, which was derived from the gain of the feature during tree splitting. The top five genes based on contribution scores were retained. Finally, given that LASSO focused on sparse linear effects (27), Boruta emphasized nonlinear importance and interactions (28), and XGBoost concentrated on gradient-based gain (29), the feature selection mechanisms of the four algorithms were complementary. Therefore, to ensure that the selected genes were not influenced by the specific biases of any single model, the VennDiagram package (v1.7.3) (30) was used to identify the genes common to all four analytical methods. These overlapping genes were defined as candidate genes potentially associated with transcriptional changes related to diabetic retinopathy and DNL.

### 2.5 Receiver operating characteristic analysis and expression verification

To assess how effectively the candidate genes distinguish between DR and control samples in the GSE221521 and GSE94019 datasets, ROC curve analysis was conducted with the pROC package (v1.18.5) (31). The curve was generated following the calculation of the false positive rate (FPR) and true positive rate (TPR). The area under the curve (AUC) was used to assess the discriminative performance of each gene, with AUC values greater than 0.7 considered indicative of acceptable predictive performance. To further assess the robustness of the candidate genes, their expression patterns were examined in both the GSE221521 and GSE94019 datasets. Genes showing statistically significant differential expression between DR and control samples and displaying consistent expression trends across both datasets were defined as key genes.

### 2.6 Construction of a nomogram

To evaluate the predictive value of the identified genes for DR, a nomogram was developed utilizing the rms package (v6.8.1) (32) based on the GSE221521 dataset. Model calibration was verified through calibration curves, and the Hosmer–Lemeshow (HL) test was conducted using the regplot package (v1.1) (33) to examine the consistency between predicted and observed outcomes. ROC curve analysis was performed to calculate the AUC for assessing the predictive performance of the nomogram (AUC > 0.7). Decision curve analysis (DCA) was performed using the ggDCA package (v1.1) (34). To ensure the model’s reliability and broader applicability, external validation was conducted using the independent dataset GSE94019. A nomogram based on the same genes was reconstructed in the validation cohort, and its predictive performance was evaluated using ROC analysis. Calibration curves and DCA were performed to further assess model accuracy and potential clinical applicability in the validation dataset. Finally, to verify whether the expression changes of the key genes identified through multidimensional screening were statistically robust, we further calculated the FDR-adjusted p-values of these genes in the original dataset (GSE221521).

### 2.7 Gene set enrichment analysis and gene-gene interaction network

To delve deeper into the potential biological roles of the identified genes in DR, GSEA was performed using the GSE221521 dataset. The background gene set “c2.cp.kegg.v7.4.symbols.gmt” was obtained from the Molecular Signatures Database (MSigDB, https://www.gsea-msigdb.org/gsea/msigdb). Correlation coefficients between each key gene and all other genes were computed using the cor function in the stats package (v4.3.3) (35), and genes were ranked according to their correlation values in descending order to generate a ranked gene list. GSEA was then performed using the clusterProfiler package (v4.15.0.3), with statistical significance criteria established as p-value below 0.05, absolute normalized enrichment score (NES) exceeding 1, and false discovery rate (FDR) under 0.25. To further investigate potential interactions between the key genes and other genes, GGI networks were analyzed using the GeneMANIA web server (http://genemania.org/).

### 2.8 Chromosome localization and molecular regulatory network analysis

To visualize the chromosomal positions of the identified genes, the RCircos package (v1.2.2) (36) was used to generate a chromosomal localization map. In addition, transcription factors (TFs) potentially regulating these genes were predicted using the hTFtarget (https://guolab.wchscu.cn/hTFtarget/) and KnockTF (http://www.licpathway.net/KnockTF/) databases. Similarly, microRNAs (miRNAs) targeting the genes were predicted using the TargetScanHuman 8.0 (https://www.targetscan.org/vert_80/), miRDB (https://mirdb.org/), and miRTarBase (https://mirtarbase.cuhk.edu.cn/~miRTarBase/miRTarBase_2025/php/index.php) databases. Only the overlapping results identified across multiple databases were retained for the construction of the regulatory network to improve prediction reliability.

### 2.9 Drug prediction, drug evaluation, and molecular docking

To identify potential compounds targeting the selected genes, the GraphBAN model was applied to predict gene–compound interaction pairs. Amino acid sequences of the selected genes (species: *Homo sapiens*) were obtained from the National Center for Biotechnology Information (NCBI) database (https://www.ncbi.nlm.nih.gov/). Simplified Molecular Input Line Entry System (SMILES) data for small molecules were obtained from the ZINC-250K compound library and used as input for the model. The predict.py script of GraphBAN was executed using pre-trained models based on the BioSNAP, BindingDB, and KIBA datasets (from the inductive mode/trained models provided in the GitHub repository) to predict potential small molecule–protein interactions. Compounds with an interaction probability (pred) greater than 0.5 and identified consistently across all three datasets were retained for further analysis. The physicochemical properties of the candidate compounds were subsequently evaluated using ADMETlab (https://admetlab3.scbdd.com/server/screening). Compounds were first screened according to small-molecule drug-likeness criteria and Lipinski’s Rule of Five (37). Further pharmacokinetic and toxicity assessments were conducted using the ADMET-AI platform (https://admet.ai.greenstonebio.com/). Compounds were excluded if they met any of the following conditions: high predicted clinical toxicity (probability > 0.25), unreliable or missing half-life predictions, low plasma protein binding (< 50%), suboptimal lipophilicity (outside the range of 1–3), or poor oral bioavailability (probability < 0.5). To evaluate binding affinity between the selected genes and compounds, the compound with the highest pred score for each gene was selected as the ligand for molecular docking. The three-dimensional structures of the proteins encoded by these genes were obtained from the Protein Data Bank (PDB) (https://www.rcsb.org). Molecular docking simulations and interaction energy analyses were performed using AutoDock Vina software (v1.2.0) (38) (binding energy < -4 kcal/mol), and docking conformations were visualized using PyMOL (v3.1.1) (39) and the online platform proteins.plus (https://proteins.plus/).

### 2.10 Molecular dynamics simulation

To further evaluate the stability and binding dynamics of the gene–compound complexes, molecular dynamics (MD) simulations were conducted using GROMACS software (v2024.4) (40). The protein was modeled using the AMBER99SB-ILDN force field, while the ligand was parameterized with the General Amber Force Field (GAFF). Ligand structures in SDF format were prepared with neutral protonation states, and atomic partial charges were assigned using the AM1-BCC method. Each system was placed in a cubic simulation box with a minimum distance of 1.0 nm from the box boundaries and solvated using TIP3P water molecules. Sodium and chloride ions were added to neutralize the system and maintain a physiological ionic strength of 0.15 M NaCl. Energy minimization was performed using the steepest descent algorithm (50,000 steps, force tolerance of 100 kJ/mol/nm). The system was subsequently equilibrated in two stages (1): an NVT ensemble for 100 ps using a V-rescale thermostat at 300 K (2); an NPT ensemble for 100 ps using the Parrinello–Rahman barostat at 1 bar. During equilibration, positional restraints (1000 kJ/mol/nm²) were applied to the heavy atoms of the protein and ligand. Production MD simulations were then conducted for 100 ns with a time step of 2 fs. All hydrogen-containing bonds were constrained using the SHAKE algorithm. Non-bonded interactions were calculated with a cutoff distance of 10 Å for van der Waals and short-range electrostatic interactions. Long-range electrostatic interactions were treated using the Particle-Mesh Ewald (PME) method with a 32 × 32 × 32 grid and fourth-order interpolation. The non-bonded pair list was updated every 10 fs with a cutoff of 10.5 Å. The stability of the complexes was evaluated by analyzing several structural and energetic parameters, including the root-mean-square deviation (RMSD) of the protein–ligand complexes, the root-mean-square fluctuation (RMSF) of protein residues, the total system energy, and the number of hydrogen bonds formed during the simulations.

### 2.11 Analysis of scRNA-seq data

Further analyses of DR-related mechanisms were conducted at the single-cell level. Specifically, the GSE165784 dataset was processed using the Seurat package. Quality control was first performed using the PercentageFeatureSet function of Seurat (v5.3.0) (41). Unless otherwise specified, all subsequent analyses in this section were conducted using the Seurat package (v5.1.0). The quality control criteria were defined as follows (1): each cell contained between 200 and 4,000 detected genes (2); the total gene expression per cell ranged from 200 to 20,000 (3); genes were expressed in at least three cells; and (4) the proportion of mitochondrial gene expression was less than 20%. Highly variable genes were identified using the FindVariableFeatures function with the parameters selection.method = “vst” and nfeatures = 3,000. The data were then scaled using the ScaleData function. Dimensionality reduction was subsequently performed using principal component analysis (PCA), and the significance of principal components (PCs) was evaluated using the JackStraw procedure. Based on the permutation test results generated by the JackStraw and ScoreJackStraw functions, PCs with stable signals were selected for downstream analysis. Cell clustering was performed in an unsupervised manner using the FindNeighbors and FindClusters functions on the selected PCs, resulting in multiple cell clusters (resolution = 0.8). To annotate cell types, the FindAllMarkers function was applied to identify marker genes highly expressed in each cluster, using the following parameters: min.pct = 0.1, logfc.threshold = 0.25, and test.use = “auc”.Cell-type annotation was performed with reference to previously reported marker genes (42). The expression patterns of the selected genes were then examined across the annotated cell types. Cell populations exhibiting relatively higher expression levels compared with other cell types were identified to provide a cellular context for the identified genes. It should be noted that, because the dataset did not include control samples (such as healthy retinal tissue or non-DR fibrous membranes), these analyses do not establish causal involvement of specific cell types in DR. Instead, they provide insight into the cellular distribution of gene expression within the fibrous membranes associated with DR.

### 2.12 Metabolic pathways, cell communication, and pseudo-time analysis

To investigate the potential biological roles of the identified cell populations, functional enrichment analysis was performed using the ReactomeGSA package (v1.14.0) (43). Given the strong association between DR and metabolic dysregulation, single-cell metabolic pathway analysis was conducted using the scMetabolism package (v0.2.1) (44). The gene expression count matrix was first extracted using the GetAssayData function, with metabolism.type set to “KEGG”. The sc.metabolism.SeuratMethod function was then applied, with “AUCell” selected to calculate metabolic pathway activity scores for each cell. Metabolic pathways were ranked according to their activity scores, and differences in the top ten metabolic pathways between cell populations were compared. In addition, the CellChat package (v1.6.1) (45) was used to investigate potential intercellular communication between the identified cell populations and other cell types. This analysis enabled the prediction of ligand–receptor interactions between these cell populations and the most abundant cell types in the dataset (p < 0.01, log_2_mean(molecule 1) ≥ 0.1, log_2_mean(molecule 2) ≥ 0.1). To further explore potential cellular differentiation processes, the selected cell populations were re-analyzed using secondary dimensionality reduction and clustering (resolution = 0.2). *Pseudotim*e trajectory analysis was then performed using the Monocle package (v2.28.0) (46) to infer potential differentiation trajectories and to observe the dynamic expression patterns of the selected genes along these trajectories.

### 2.13 RT-qPCR

Peripheral blood specimens were obtained from five individuals diagnosed with DR and five healthy participants at the Ophthalmology Department of Zhengzhou University’s First Affiliated Hospital. Total RNA was subsequently extracted from these samples. Reverse transcription quantitative PCR (RT-qPCR) was performed on a QuantStudio 3 instrument to validate gene expression levels, with three technical replicates included for each sample. Glyceraldehyde-3-phosphate dehydrogenase (*GAPDH*) served as the endogenous reference gene. Relative expression levels of the selected genes in peripheral blood samples from patients with DR and healthy controls were calculated using the 2^−ΔΔCt^ method, and differences between the two groups were evaluated using Student’s t-test. The absence of a diabetic without retinopathy (DM) control group in this RT-qPCR validation limits the ability to determine whether the observed expression differences are specific to DR or reflect diabetes more generally. To partially address this limitation, additional in silico validation was performed using the GSE221521 dataset, which includes samples from patients with DR (*n* = 69) and individuals with diabetes without retinopathy (*n* = 74).

### 2.14 Statistical analysis

Bioinformatics and statistical evaluations were executed using R software (v4.2.2). Given the non-normal distribution of sequencing data, the Wilcoxon rank-sum test, a non-parametric method, was employed to compare transcriptomic data between DR patients and control subjects. Statistical significance was defined as *p* < 0.05 across all analyses.

## 3 Results

### 3.1 The 8 DNL signature genes associated with DR

**Figure 1:**
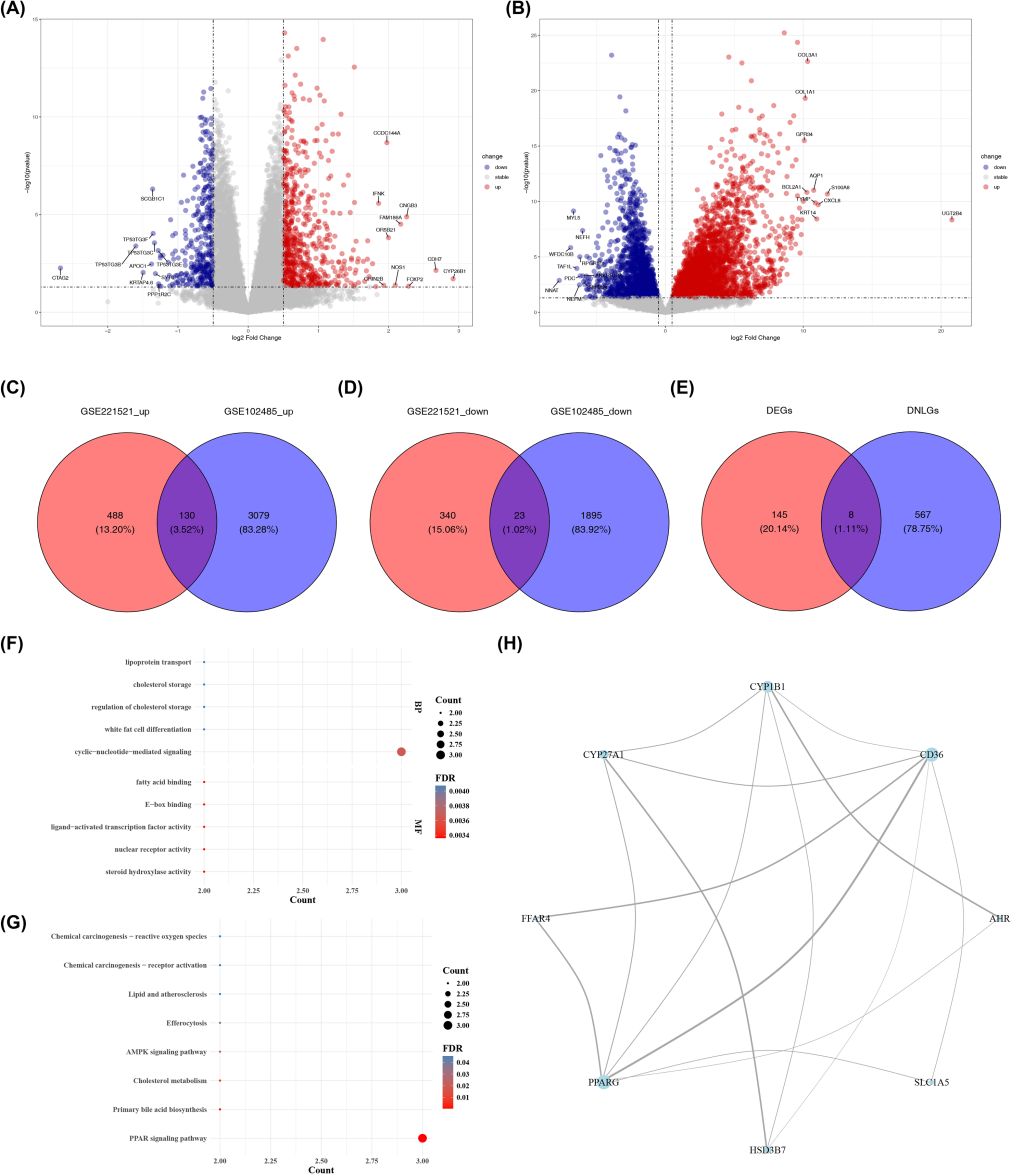
Acquisition, functional enrichment, and network construction of directional genes. **(A)** Volcano plot displaying 981 differentially expressed genes (DEGs) from the GSE221521 dataset, with 618 genes significantly upregulated and 363 genes downregulated in the DR group. **(B)** Volcano plot illustrating 5,127 DEGs identified in the GSE102485 dataset, of which 3,209 were upregulated, and 1,918 were downregulated. **(C)** Venn diagram illustrating the intersection of 618 upregulated genes from GSE221521 and 3209 upregulated genes from GSE102485. **(D)** Venn diagram illustrating the overlapping genes between 363 downregulated genes from GSE221521 and 1918 downregulated genes from GSE102485. **(E)** Venn diagram presents the overlapping genes between those with consistent expression patterns in GSE221521 and GSE102485, and *de novo* lipogenesis (DNL)-related genes. **(F)** Bubble plot of Gene Ontology (GO) enrichment analysis **(G)** Bubble plot of Kyoto Encyclopedia of Genes and Genomes (KEGG) enrichment analysis showing the top five pathways ranked by adjusted p-values. **(H)** Protein–protein interaction (PPI) network of directional genes. Each dot in the volcano plot represents an individual gene. Red dots signify upregulated genes, blue dots represent downregulated genes, and gray dots indicate genes with no statistically significant changes. In the PPI network, the size of each node represents the degree of protein connectivity. Lines indicate interactions between proteins, and thicker lines represent stronger interaction confidence.

Differential expression analysis was conducted separately in two independent datasets. In the GSE221521 dataset (peripheral blood), comparison between DR patients and healthy controls identified 981 DEGs, including 618 upregulated and 363 downregulated genes (**Figure 1A**). In the GSE102485 dataset (retinal fibrovascular membranes), comparison between patients with PDR and normal retinal samples identified 5,127 DEGs, including 3,209 upregulated and 1,918 downregulated genes (**Figure 1B**). To identify genes showing consistent dysregulation across both peripheral blood and retinal tissue—and thereby reduce tissue-specific bias—the upregulated and downregulated genes from the two datasets were intersected separately. This analysis identified 130 commonly upregulated genes and 23 commonly downregulated genes (**Figures 1C, D**). These 153 genes were further intersected with the 575 genes associated with DNL, resulting in eight overlapping genes with DNL-related expression signatures. These genes included *CD36*, *CYP27A1*, *AHR*, *SLC1A5*, *CYP1B1*, *HSD3B7*, *PPARG*, and *FFAR4* (**Figure 1E**). To explore the potential biological functions of these eight DNL-related genes in DR, we performed GO and KEGG enrichment analyses, and all results were screened and presented based on the adjusted significance threshold (*p*adj < 0.05). The GO enrichment analysis results showed that these genes were primarily involved in biological processes such as lipid metabolism, inflammation, and cell signal transduction. In terms of biological processes (BP), the genes were significantly enriched in pathways including the regulation of cholesterol storage, lipoprotein transport, and white adipocyte differentiation. Regarding molecular functions (MF), they were enriched in functions such as nuclear receptor activity, ligand-activated transcription factor activity, and fatty acid binding (**Figure 1F**; **Supplementary Table 2**). KEGG pathway enrichment analysis further revealed that these genes were significantly involved in eight pathways, mainly including the PPAR signaling pathway, AMPK signaling pathway, primary bile acid biosynthesis, and cholesterol metabolism. These pathways are closely related to energy metabolism, lipid homeostasis, and inflammatory responses, suggesting that these genes might participate in the pathological process of DR through these pathways (**Figure 1G**; **Supplementary Table 2**). Protein–protein interaction (PPI) analysis revealed interactions among the proteins encoded by these genes, forming a network with 16 interaction edges. CD36, PPARG, and CYP1B1 interacted with five or more other proteins, suggesting relatively central positions within the interaction network (**Figure 1H**).

### 3.2 *AHR* and *SLC1A5* associated with DNL signatures in DR

**Figure 2:**
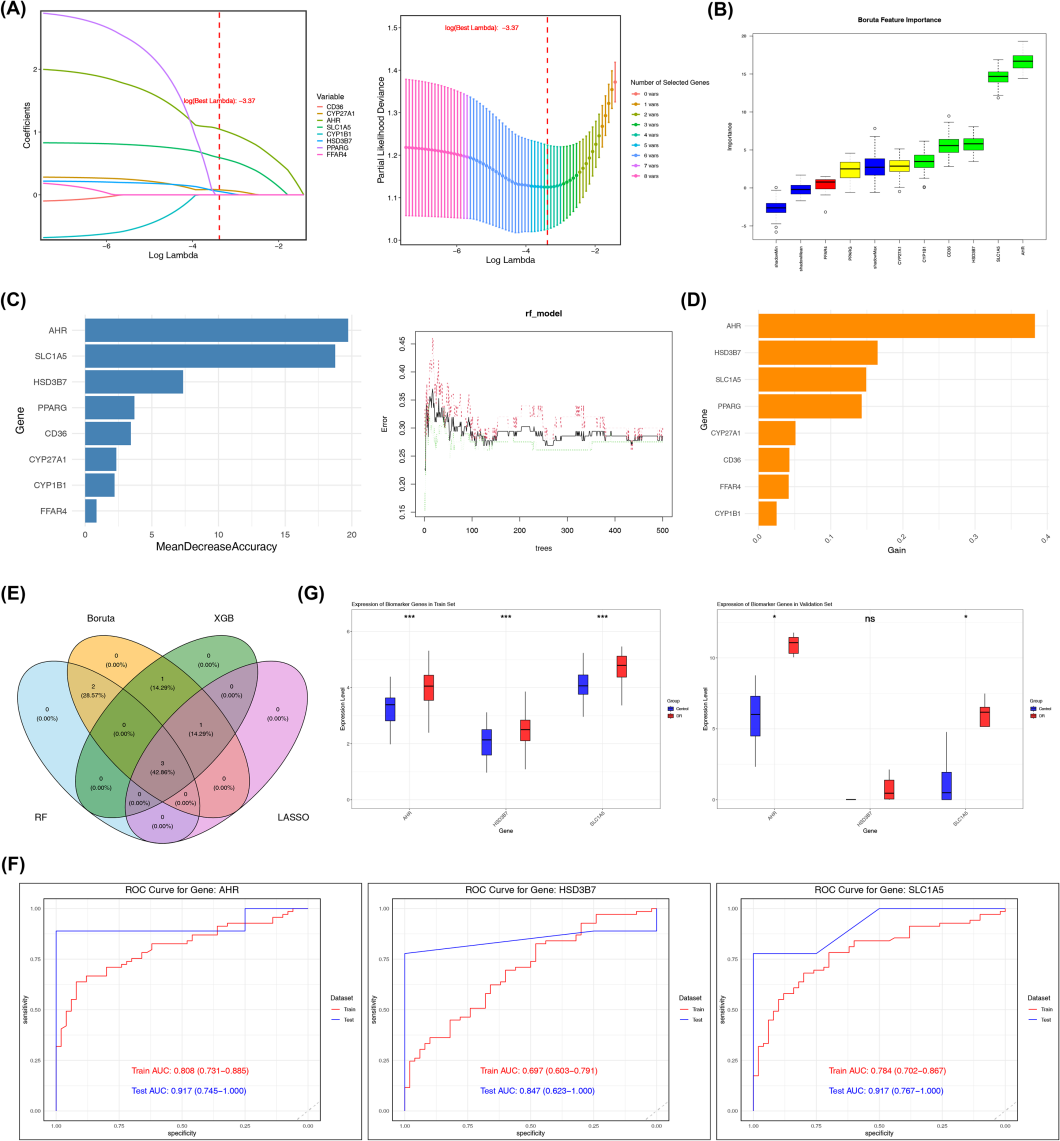
Machine learning–based screening of candidate genes associated with DR. **(A)** Least absolute shrinkage and selection operator (LASSO) regression analysis identified 4 genes with non-zero coefficients (not penalized to zero) at log (best lambda) = -3.37. **(B)** Under the Boruta algorithm, genes marked in green were determined as important factors, with a total of 5 genes. **(C)** Based on the optimal mtry value, a random forest model was built, and the top 5 genes by feature importance were retained for further analysis. **(D)** In the XGBoost algorithm, the top 5 genes were retained based on the feature importance plot. **(E)** Overlap of feature genes identified by the 4 machine learning methods. **(F)** Receiver operating characteristic (ROC) curves of the training set (GSE221521) and validation set (GSE94019). Genes with an area under the curve (AUC) > 0.7 were considered to have good diagnostic performance. **(G)** Expression validation in the training set (GSE221521) and validation set (GSE94019). Ns = no significance; * = *p* < 0.05; *** = *p* < 0.001.

To further prioritize genes of potential relevance, four machine learning algorithms were applied. LASSO regression analysis showed that when log (best lambda) was −3.37, the coefficients of *CYP27A1*, *AHR*, *SLC1A5*, and *HSD3B7* remained non-zero, indicating that these genes were retained by the model (**Figure 2A**). In the Boruta analysis, the genes identified as important features included *AHR*, *SLC1A5*, *HSD3B7*, *CD36*, and *CYP1B1* (**Figure 2B**). Similarly, a random forest model constructed with the optimal parameter mtry = 3 identified the top five genes as *AHR*, *SLC1A5*, *HSD3B7*, *PPARG*, and *CD36* (**Figure 2C**). In the XGBoost analysis, the top five genes ranked by contribution score using the xgb_importance function were *AHR*, *HSD3B7*, *SLC1A5*, *PPARG*, and *CYP27A1* (**Figure 2D**). By intersecting the results of the four machine learning approaches, three overlapping genes were identified as candidate genes (**Figure 2E**). In both the GSE221521 dataset and the independent validation dataset GSE94019, *AHR* and *SLC1A5* showed AUC values greater than 0.750 (**Figure 2F**). In addition, both genes exhibited significantly higher expression levels in DR samples compared with control samples in the two datasets (**Figure 2G**). These findings suggest that *AHR* and *SLC1A5* may represent candidate genes associated with DNL-related transcriptional alterations in DR. To further verify the robustness of the screening results, we examined the FDR-adjusted p-values of *AHR* and *SLC1A5* in the original training set (GSE221521). The results showed that, even under a more stringent significance threshold (padj < 0.05), the differential expression of these two genes between the DR group and the control group remained highly significant. Specifically, the log2FC for *AHR* was 0.784, with a padj value of 9.89e^-09^; the log2FC for *SLC1A5* was 0.552, with a padj value of 1.55e^-05^. These results confirmed that our screening findings were statistically robust and were not false positives resulting from multiple hypothesis testing (**Supplementary Table 3**).

### 3.3 The nomogram integrating *AHR* and *SLC1A5* with good performance in predicting DR prevalence

**Figure 3:**
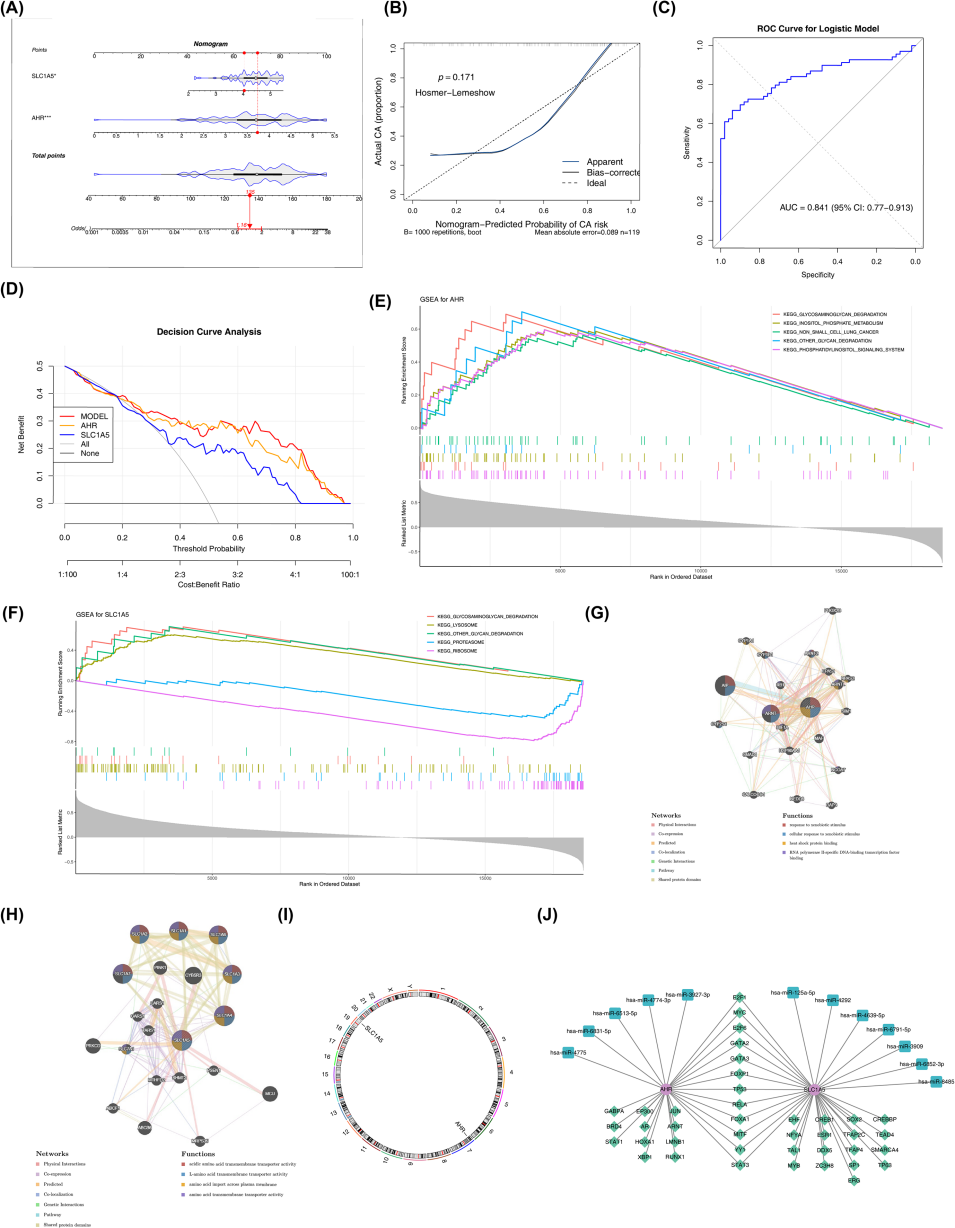
Molecular characterization of candidate genes and construction of the nomogram model. **(A)** In the nomogram, DR prevalence increases with the elevation of total points. **(B)** Calibration curve of the nomogram model. The x-axis represents the nomogram-predicted probability of DR, and the y-axis the actual probability of DR. The black dashed line stands for perfect prediction, the blue solid line for the entire cohort, and the black solid line for the observed nomogram performance after bias correction with 1,000 bootstrap repetitions **(C)** ROC curves of nomogram, and nomogram with an AUC > 0.7 were considered to have good performance. **(D)** Decision curve analysis (DCA) of the nomogram. The gray curve (labeled ‘all’) denotes the net benefit of treating all patients, and the black curve (labeled ‘none’) represents the net benefit of providing no treatment to any patient. **(E, F)** Gene set enrichment analysis (GSEA) of *AHR* **(E)** and *SLC1A5* **(F)**, displaying the top 5 pathways ranked by the absolute values of normalized enrichment score (NES). The X-axis represents genes sorted in descending order based on gene correlation. **(G, H)** Gene–gene interaction network of *AHR* **(G)** and *SLC1A5* **(H)**, illustrating their respective interaction modes and associated pathways with related genes. **(I)** Chromosomal localization of the candidate genes. **(J)** Regulatory network showing candidate genes (purple), microRNAs (blue), and transcription factors (green).

The nomogram further demonstrated the predictive potential of the selected genes for DR prevalence. Specifically, it indicated that the predicted probability of DR increased as the total score increased (**Figure 3A**). Calibration curve analysis was performed to assess the predictive reliability of the nomogram, and the results showed good agreement between the bias-corrected predicted probabilities of the DR diagnostic nomogram and the ideal reference line. The HL test yielded a p-value of 0.171, further confirming no significant deviation between model-predicted and actual observed outcomes. ROC curve analysis was conducted to evaluate the discriminatory ability of the nomogram, which yielded an AUC of 0.841. (**Figures 3B, C**). DCA showed that the nomogram provided a positive net benefit across a range of threshold probabilities and outperformed both the “treat-all” and “treat-none” strategies, as well as models based on individual genes alone (**Figure 3D**). Together, these findings suggest that the nomogram constructed from *AHR* and *SLC1A5* may have potential value for predicting DR risk. To assess the generalizability of the model and reduce the possibility of overly optimistic performance estimates, external validation was performed using the independent dataset GSE94019. The nomogram was reconstructed using *AHR* and *SLC1A5* expression data in the validation cohort (**Supplementary Figure 1A**). Calibration curves revealed high consistency between predicted and observed probabilities (HL test, *p* = 0.182) (**Supplementary Figure 1B**). The model maintained good predictive performance in the external dataset, with an AUC of 0.889 (**Supplementary Figure 1C**). In the validation cohort, DCA confirmed that the nomogram provided a positive net clinical benefit across a range of threshold probabilities (**Supplementary Figure 1D**). These results suggest that the nomogram based on *AHR* and *SLC1A5* demonstrates stable predictive performance across datasets.

### 3.4 Key genes significantly involved in DR pathogenesis-related pathways

Functional enrichment analysis revealed that selected genes were significantly enriched in several biological pathways related to metabolism, inflammation, cellular signaling, and vascular regulation. These pathways included the insulin signaling pathway, Toll-like receptor (TLR) signaling pathway, and focal adhesion, among others (**Figures 3E, F**; **Supplementary Table 4**). In the GGI network, *AHR* and its associated genes were primarily connected through predicted interactions, physical interactions, and shared protein domains. Among these genes, *AHR* showed close associations with *AIP* and *ARNT*, which participated in biological processes such as response to xenobiotic stimulus and cellular response to xenobiotic compounds. In contrast, *SLC1A5* interacted with other genes mainly through shared protein domains, co-expression relationships, and physical interactions. *SLC1A5* showed strong similarity with members of the *SLC1* transporter family (*SLC1A1*, *SLC1A2*, *SLC1A3*, *SLC1A4*, *SLC1A6*, and *SLC1A7*) and was associated with biological functions such as acidic amino acid transmembrane transporter activity (**Figures 3G, H**). These interaction networks provide additional context for understanding the regulatory relationships surrounding the identified genes.

### 3.5 Targeted regulatory network of *AHR* and *SLC1A5*

Chromosomal localization analysis showed that *AHR* and *SLC1A5* are located on chromosomes 7 and 19, respectively (**Figure 3I**). These findings provide basic genomic information that may be relevant for further genetic investigations. Using the hTFtarget and KnockTF databases, 23 TFs potentially regulating *AHR* and 29 TFs potentially regulating *SLC1A5* were identified. Notably, 12 TFs were shared between the two genes. For example, E2F1, MYC, and E2F6 were predicted to regulate both *AHR* and *SLC1A5*. Similarly, predictions from the TargetScanHuman 8.0, miRDB, and miRTarBase databases identified five microRNAs potentially targeting *AHR* and seven targeting *SLC1A5*, including hsa-miR-3927-3p (*AHR*) and hsa-miR-125a-5p (*SLC1A5*) (**Figure 3J**). These predicted regulatory relationships provide a preliminary framework for understanding the potential transcriptional and post-transcriptional regulation of these genes.

### 3.6 Potential of key genes as candidate molecular targets

**Figure 4:**
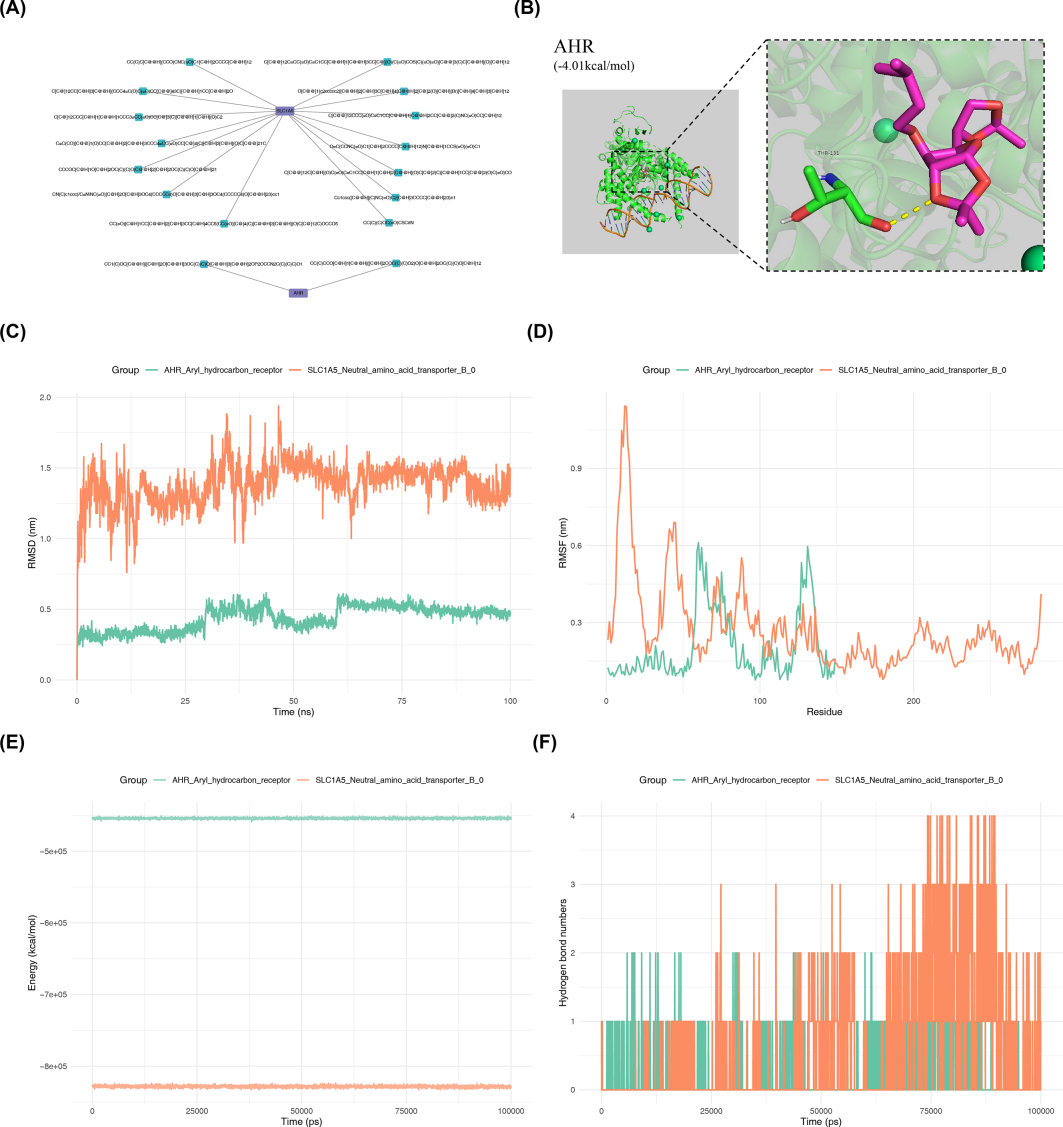
Compound prediction and evaluation for candidate genes. **(A)** Key gene-core compound pairs obtained from predictions by the GraphBAN model (integrating BioSNAP, BindingDB, and KIBA datasets) and evaluation using Lipinski’s Rule of Five and drug-likeness. **(B)** Molecular docking of *AHR* with compounds showing the highest average prediction (pred) scores. Purple represents the compound structure, green represents protein residues interacting with the compound, and yellow represents hydrogen bonds. **(C)** Root mean square deviation (RMSD) of the complex; smaller fluctuations indicate more stable binding between the protein and drug. **(D)** Root mean square fluctuation (RMSF) of the complex; larger fluctuations indicate greater flexibility of protein amino acids. **(E)** Total energy of the complex; lower energy with smaller fluctuations indicates higher stability. **(F)** Number of hydrogen bonds in the complex; more hydrogen bonds indicate a more stable binding.

GraphBAN analysis predicted a large number of potential compound interactions for the selected genes. For *AHR*, 139,676, 5,522, and 605 compounds with interaction probabilities greater than 0.5 were identified in the BioSNAP, BindingDB, and KIBA datasets, respectively. Intersection analysis across the three datasets identified six shared compounds. For *SLC1A5*, 12,499, 210,860, and 282 compounds with interaction probabilities greater than 0.5 were obtained from the BioSNAP, BindingDB, and KIBA datasets, respectively, and 49 compounds were identified in the intersection set. Subsequent filtering based on Lipinski’s Rule of Five retained all six compounds associated with *AHR*, while 47 compounds targeting *SLC1A5* remained after screening. Further evaluation of drug-like properties identified two core compounds targeting *AHR* and fourteen targeting *SLC1A5* (**Figure 4A**), with detailed information summarized in **Table 1**. The target protein structures were obtained from the PDB database, with the crystal structure with the ID 5NJ8 used for *AHR* and the crystal structure with the ID 5LLM used for *SLC1A5*. The docking box center coordinates were set as follows: *AHR* (*X* = 6.281, *Y* = 30.271, *Z* = 216.216); *SLC1A5* (*X* = -45.142, *Y* = 444.082, *Z* = -4.382), to ensure coverage of the potential active regions. Molecular docking analysis was then performed using the compounds with the highest predicted interaction scores. The binding energy between *AHR* and the compound CC(C)CCO[C@H]1C@@HO[C@@H]2OC(C)(C)O[C@H]12 was −4.01 kcal/mol (**Figure 4B**). For *SLC1A5*, the binding energy with the compound C[C@@]12CCC(=O)C=C1CC[C@@H]1[C@@H]2CC[C@@]2(C)NC(=O)CC[C@H]12 was −6.09 kcal/mol. Both binding energies were lower than the preset screening threshold (< -4 kcal/mol), indicating potential interactions between these compounds and the target proteins. To further assess the stability of these complexes, molecular dynamics simulations were conducted. The RMSD of the *SLC1A5* complex remained within the range of 0.25–0.60 nm during the 0–100 ns simulation period, indicating relatively stable structural behavior. In contrast, the *AHR* complex showed RMSD values mainly ranging from 0.75 to 1.75 nm, with comparatively larger fluctuations (**Figure 4**). Analysis of RMSF showed that the *SLC1A5* complex exhibited higher flexibility in the 0–50 amino acid residue region, which may correspond to a potential ligand-binding region. In other regions, RMSF values generally remained within 0–0.6 nm. For the *AHR* complex, overall RMSF values were relatively low, with somewhat higher fluctuations observed mainly in the 50–70 and 120–150 amino acid residue regions (**Figure 4D**). Both complexes exhibited relatively low and stable total system energy during the simulations, with the *SLC1A5* complex displaying slightly lower energy than the *AHR* complex (**Figure 4E**). In addition, hydrogen bond analysis showed that the *AHR* complex maintained 1–2 hydrogen bonds, whereas the *SLC1A5* complex formed 1–4 hydrogen bonds during the simulation period, which is consistent with the molecular docking results (**Figure 4F**). It was noted that the above results were all based on computer simulation predictions and only suggested that the candidate compounds might have potential binding abilities with the target proteins. Their actual interactions and biological effects still need to be verified through subsequent wet experiments (such as surface plasmon resonance, cellular thermal shift assays, etc.).

**Table 1:** Key gene-core compound pairs.

Gene	ZINC ID	SMILES	MW	nRot	nHA	nHD	LogP	BioavailabilityMa	ClinTox	Half_Life_Obach	Lipophilicity_AstraZeneca	PPBR_AZ	bindingdb	biosnap	kiba
*AHR*	ZINC36370239	CC1(C)OC[C@@H]([C@H]2O[C@@H]3OC(C)(C)O[C@@H]3[C@@H]2OP2OCCN2C(C)(C)C)O1	405.19	4	8	0	2.182	0.926	0.1548	64.0019	1.4416	73.1746	0.602	0.554	0.883
*AHR*	ZINC4745227	CC(C)CCO[C@H]1[C@@H]([C@H]2COC(C)(C)O2)O[C@@H]2OC(C)(C)O[C@H]12	330.2	5	6	0	3.136	0.977	0.065	32.1276	1.848	68.0627	0.524	0.548	0.956
*SLC1A5*	ZINC35422984	CN(C)c1ccc(/C=N\NC(=O)[C@H]2O[C@H]3OC4(CCCCC4)O[C@@H]3[C@H]3OC4(CCCCC4)O[C@@H]23)cc1	499.27	5	9	1	4.883	0.8872	0.216	81.3614	2.8594	88.6826	0.913	0.667	0.607
*SLC1A5*	ZINC28632339	C[C@@]12C=CC(=O)C=C1CC[C@@H]1[C@@H]3CC[C@](O)(C(=O)COS(C)(=O)=O)[C@@]3(C)C[C@@H](O)[C@H]12	438.17	4	7	2	1.789	0.7976	0.0947	41.0365	1.1079	73.8074	0.681	0.611	0.874
*SLC1A5*	ZINC175263742	CC(C)C[C@@H](CCO)CNC(=O)C1[C@H]2CCCC[C@@H]12	267.22	8	3	2	3.392	0.8809	0.0964	29.4464	2.4549	67.0212	0.513	0.636	0.789
*SLC1A5*	ZINC136797318	C[C@@]12C[C@H](O)C(=O)C=C1CC[C@H]1[C@H]2[C@@H](O)C[C@]2(C)[C@@H]1CC[C@@]2(O)C(=O)CO	378.2	2	6	3	1.798	0.8074	0.0922	7.6478	1.0507	61.5987	0.659	0.602	0.88
*SLC1A5*	ZINC245452500	O=C(CCNC(=O)C1[C@H]2CCCC[C@@H]12)N[C@@H]1CCS(=O)(=O)C1	328.15	7	6	2	-0.272	0.6631	0.1225	19.3858	1.5866	53.5743	0.644	0.618	0.762
*SLC1A5*	ZINC1591781	CC(C)(C)OC(=O)CSC#N	173.05	4	3	0	1.749	0.6319	0.0036	7.5583	1.8554	75.1111	0.795	0.571	0.846
*SLC1A5*	ZINC100296983	O[C@@]1(c2ccccc2)[C@@H]2[C@H]3C[C@H]4[C@@H]2[C@]2(O)[C@H](Br)[C@H]4[C@H]3[C@H]12	332.04	1	2	2	2.367	0.8972	0.0048	14.3998	2.0368	61.0023	0.769	0.565	0.999
*SLC1A5*	ZINC40164440	C[C@]12CC[C@H]3[C@@H](CCC4=C(O)C(=O)CC[C@@]43C)[C@@H]1CC[C@@H]2O	304.2	0	3	1	3.084	0.5972	0.0099	4.4844	2.7429	81.6168	0.598	0.565	0.517
*SLC1A5*	ZINC118917252	CC(=O)[C@H]1CC[C@@H]2[C@H]3CC[C@H]4CC5(CC(=O)[C@]4(C)[C@@H]3[C@@H](O)C[C@]12C)OCCO5	390.24	1	5	1	2.542	0.9179	0.0723	43.0441	2.479	75.6411	0.584	0.604	0.594
*SLC1A5*	ZINC174555603	Cc1csc([C@@H](C)NC(=O)C2[C@H]3CCCC[C@@H]23)n1	264.13	4	3	1	3.063	0.96	0.044	5.503	2.5892	68.2187	0.647	0.505	0.684
*SLC1A5*	ZINC4983525	C[C@@]12CCC(=O)C=C1CC[C@@H]1[C@@H]2CC[C@@]2(C)NC(=O)CC[C@H]12	301.2	0	3	1	3.342	0.7959	0.0263	0.9214	2.0093	73.2219	0.593	0.598	0.541
*SLC1A5*	ZINC2132389	C=C(CO)[C@@]1(O)CC[C@@H]2[C@@H]3CCC4=CC(=O)CC[C@]4(C)[C@H]3[C@H](O)C[C@@]21C	360.23	2	4	3	2.036	0.64	0.043	2.2533	2.1023	76.2436	0.621	0.629	0.733
*SLC1A5*	ZINC25541794	C[C@]12CCC[C@H]1[C@@H]1CCC3=CC(=O)CC[C@]3(C)[C@@H]1[C@H](O)C2	288.21	0	2	1	3.635	0.6829	0.0111	10.989	2.9241	78.0827	0.617	0.59	0.679
*SLC1A5*	ZINC229680017	CCCOC[C@H]1O[C@H]2OC(C)(C)O[C@@H]2[C@H]2OC(C)(C)O[C@@H]21	302.17	4	6	0	2.151	0.9755	0.0382	40.693	1.0899	55.2694	0.53	0.64	0.778

SMILES: Simplified Molecular-Input Line-Entry System representation of small molecule structures; MW: Molecular Weight; nRot: Number of rotatable bonds; nHA: Number of hydrogen bond acceptors; nHD: Number of hydrogen bond donors; LogP: Octanol-water partition coefficient; BioavailabilityMa: Predicted oral bioavailability; ClinTox: Predicted clinical toxicity; Half_Life_Obach: Predicted half-life (Obach model); Lipophilicity_AstraZeneca: Predicted lipophilicity value (AstraZeneca model); PPBR_AZ: Plasma protein binding rate (AstraZeneca model); bindingdb: Interaction probability predicted by the BindingDB model; biosnap: Interaction probability predicted by the BioSNAP model; kiba: Interaction probability predicted by the KIBA model.

### 3.7 Single-cell landscape associated with DR

**Figure 5:**
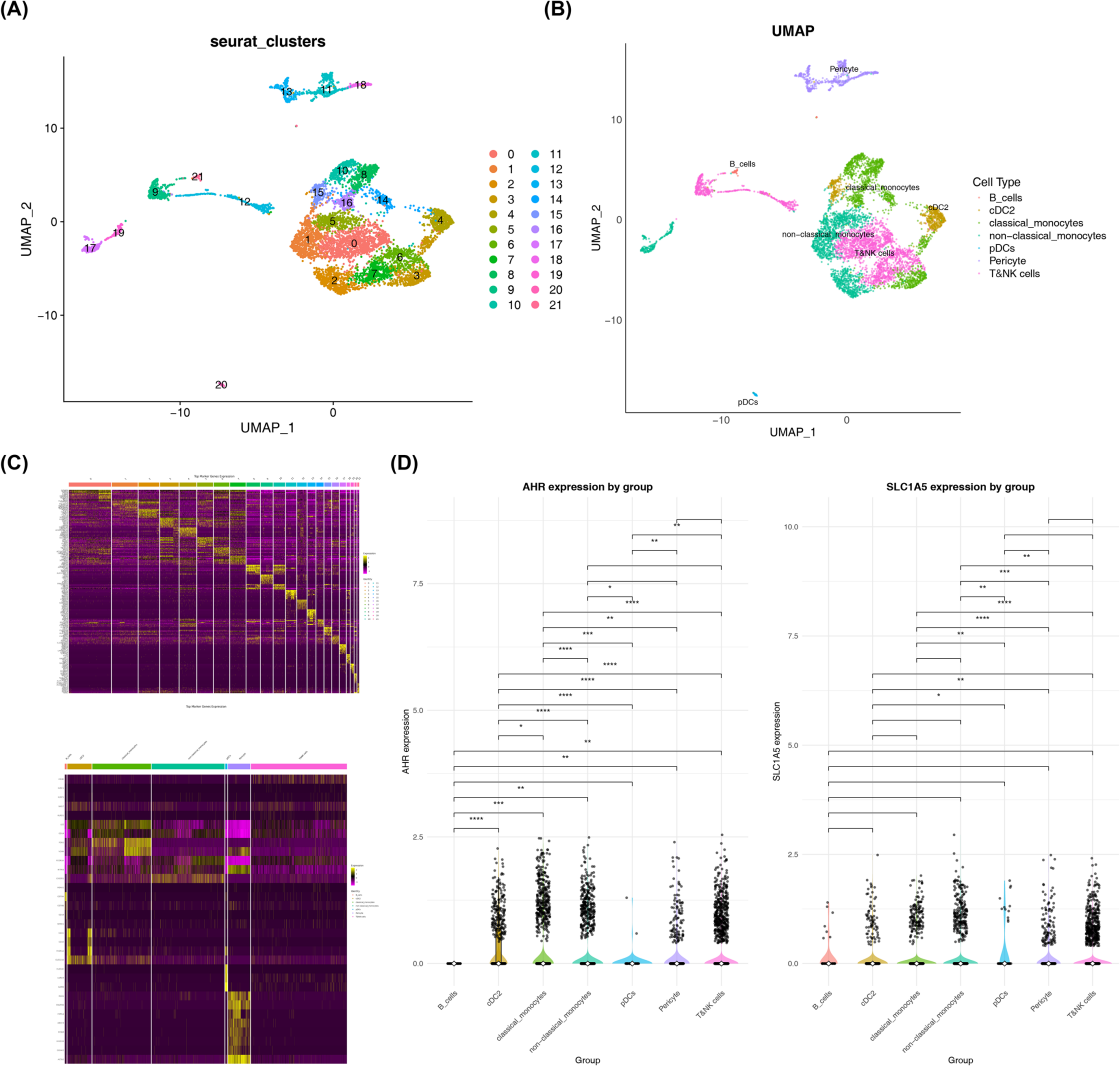
Single-cell transcriptomic analysis of DR samples. **(A, B)** Uniform Manifold Approximation and Projection (UMAP) clustering plots of single-cell data before and after cell-type annotation. Each point represents a single cell and is colored according to cluster or annotated cell type. **(C)** Heatmap of marker gene expression patterns across cell clusters. **(D)** Comparison of the expression differences of key genes across cell types. * = p < 0.05; ** = p < 0.01; *** = p < 0.001; **** = p < 0.0001.

The GSE165784 dataset initially contained 7,971 cells and 20,913 genes before quality control. After filtering, 6,883 cells and 20,913 genes were retained for further analysis (**Supplementary Figures 2A, B**). The top 3,000 highly variable genes, including *MMP9*, *CLU*, and *IGHG1*, were identified (**Supplementary Figure 2C**). Dimensionality reduction analysis showed that the cumulative variance explained by the top 20 PCs was 0.8309738, and the signal plateaued after the twentieth component; therefore, the top 20 PCs were selected for downstream analyses (**Supplementary Figure 2D**). Using a clustering resolution of 0.8, unsupervised clustering identified 22 clusters, which were subsequently annotated into seven cell types: T and natural killer (NK) cells, classical monocytes, B cells, conventional dendritic cell type 2 (cDC2), non-classical monocytes, plasmacytoid dendritic cells (pDCs), and pericytes. The validity of these cell type annotations was confirmed through examination of characteristic gene markers (**Figures 5A–C**). Notably, *AHR* showed differential expression across several cell populations, including cDC2, classical monocytes, and other cell types (**Figure 5D**). Based on these expression patterns, cDC2 and classical monocytes were identified as cell populations showing relatively higher *AHR* expression. It is important to emphasize that this observation reflects the cellular distribution of *AHR* expression within the fibrous membrane microenvironment of DR, rather than establishing these cell types as disease-driving or “key” cell populations in DR pathogenesis. Because control samples were not available in this dataset, the single-cell analysis provides descriptive cellular context for gene expression patterns rather than direct causal inference regarding disease mechanisms.

### 3.8 Elucidating the communication and differentiation of involved DR cells

**Figure 6:**
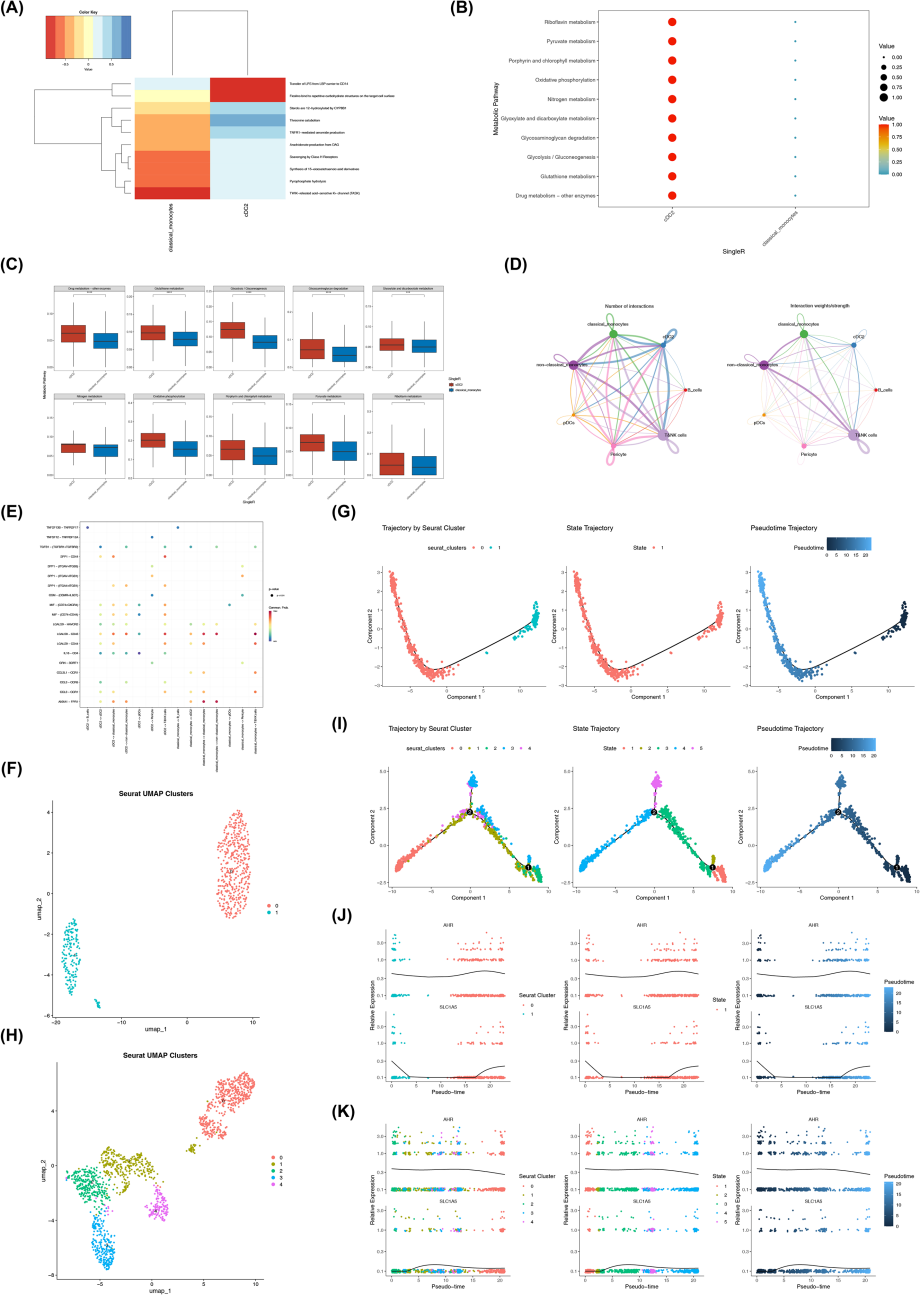
Single-cell analysis of cell populations showing relatively high candidate gene expression. **(A)** Top 10 pathways showing the greatest differences between cDC2 and classical monocytes (cell populations showing relatively higher *AHR* expression). Colors closer to red indicate higher enrichment levels. **(B)** Top 10 metabolic pathways ranked by pathway scores in the two cell populations. Dot size represents the proportion of cells, and colors closer to red indicate higher pathway activity. **(C)** Differences in activity scores of the top 10 metabolic pathways between the two cell populations. **(D)** Intercellular communication network, where each color represents a cell type; thicker lines between cells indicate stronger interactions. **(E)** Ligand-receptor pairs involved in cell communication; the redder the circle, the higher the probability of involvement. **(F)** Secondary clustering of conventional dendritic cell type 2 (cDC2). **(G)** Pseudo-time analysis of cDC2 cells, with dark blue indicating the early differentiation stage and light blue indicating the late differentiation stage. Each dot represents a single cell. **(H)** Secondary clustering of classical monocytes **(I)** *Pseudotim*e trajectory analysis of classical monocytes. **(J)** Dynamic expression patterns of candidate genes during cDC2 differentiation, represented by black curves. **(K)** Expression dynamics of candidate genes along the classical monocyte differentiation trajectory. *** = *p* < 0.001; **** = *p* < 0.0001.

Among the ten pathways with the largest differences in enrichment levels, cDC2 showed elevated enrichment primarily in two pathways: transfer of lipopolysaccharide (LPS) from the LBP carrier to CD14 and binding of ficolins to repetitive carbohydrate structures on the target cell surface. In contrast, classical monocytes showed enrichment in a broader range of pathways, including TWIK-related acid-sensitive K^+^ channel (TASK) signaling, pyrophosphate hydrolysis, synthesis of 15-eicosatetraenoic acid derivatives, and scavenging by class H receptors (**Figure 6A**). Analysis of metabolic activity showed that the top ten pathways with the highest metabolic scores included riboflavin metabolism, pyruvate metabolism, and porphyrin, among others. These pathways exhibited relatively high activity in cDC2. Moreover, compared with classical monocytes, all of these pathways showed significantly higher activity in cDC2 (**Figures 6B, C**). Together, these findings suggest that cDC2 and classical monocytes display distinct pathway enrichment patterns and metabolic activity profiles, indicating potential functional differences between these cell populations in the studied cellular environment. Analysis of cell–cell communication revealed that cDC2, T and NK cells, pericytes, and non-classical monocytes accounted for the majority of communication events with other cell types. In terms of communication strength, non-classical monocytes showed the strongest interactions with classical monocytes and T and NK cells, followed by interactions between T and NK cells and classical monocytes. In addition, cDC2 primarily communicated with T and NK cells, classical monocytes, and non-classical monocytes (**Figure 6D**). Ligand–receptor analysis suggested that LGALS9–CD45 and ANXA1–FPR1 interactions may participate in communication among classical monocytes, between classical monocytes and non-classical monocytes, and between cDC2 and classical monocytes (**Figure 6E**). These predicted interactions provide potential insights into signaling mechanisms that may contribute to cellular coordination within the fibrous membrane microenvironment. To further explore potential differentiation processes, *pseudotim*e trajectory analysis was conducted. At a clustering resolution of 0.2, cDC2 cells were re-clustered into two subclusters. Subcluster 1 corresponded to early-stage differentiation, whereas subcluster 0 represented cells mainly distributed in the middle-to-late differentiation stages (**Figures 6F, G**). Similarly, classical monocytes were re-clustered into five subclusters. Subcluster 2, subcluster 1, subclusters 3 and 4, and subcluster 0 corresponded to the early, early-to-middle, middle, and late stages of classical monocyte differentiation, respectively (**Figures 6H, I**). The expression dynamics of the selected genes showed distinct patterns along these trajectories. *AHR* displayed a similar trend in both cell populations: its expression gradually decreased during early differentiation, followed by a slight increase at the middle-to-late stage and a subsequent decrease. In contrast, *SLC1A5* showed different patterns between the two cell types. In cDC2, *SLC1A5* expression initially decreased, remained relatively stable from the early-to-middle to the middle-to-late stage, and then increased at the late stage. In classical monocytes, *SLC1A5* expression showed a modest increase at the early-to-middle stage, followed by a gradual decrease (**Figures 6J, K**). These dynamic expression patterns suggest that *AHR* and *SLC1A5* may be associated with cellular differentiation processes and functional maturation of these cell populations.

### 3.9 Verification of RT-qPCR expression level

**Figure 7:**
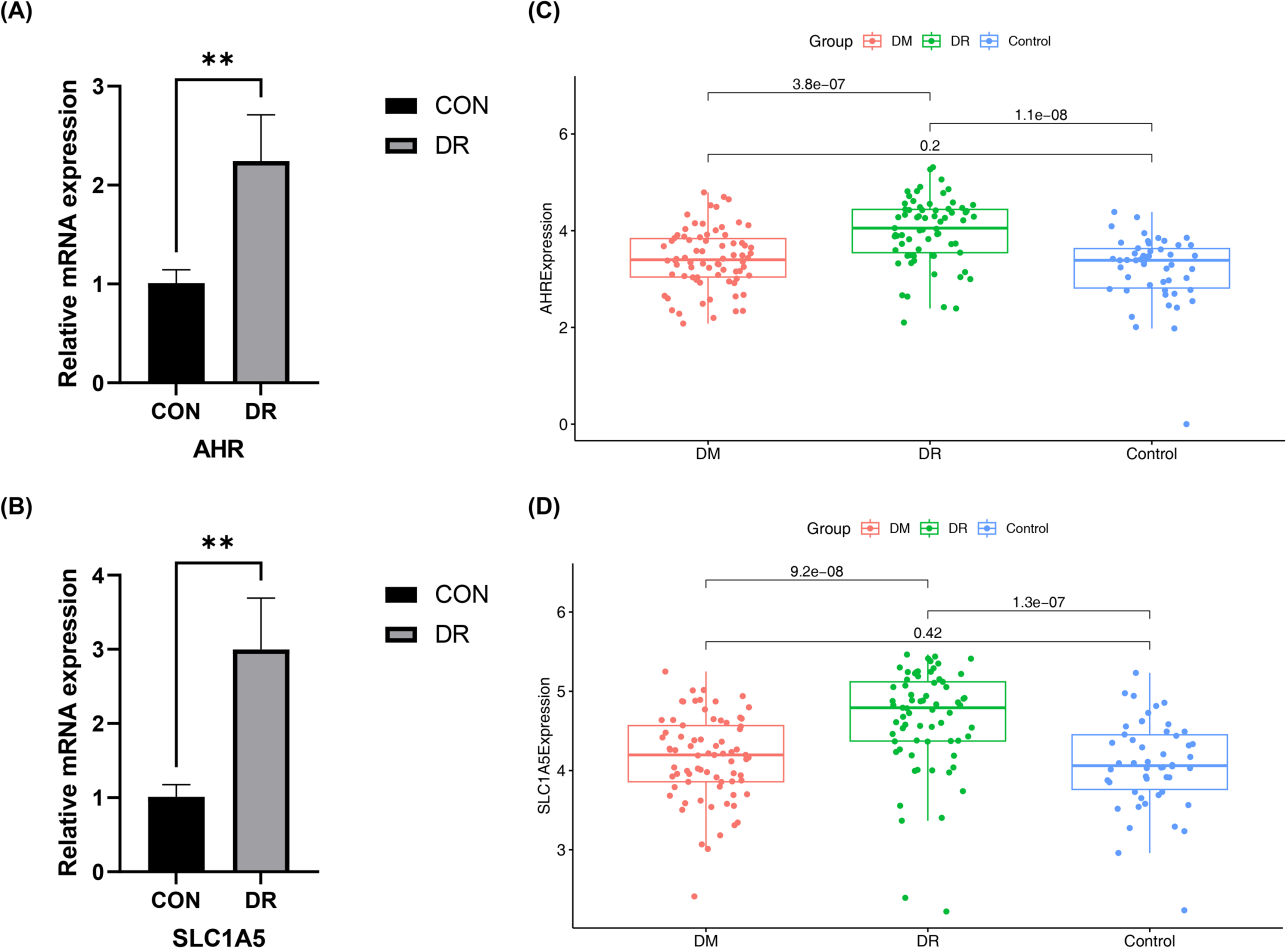
Validation of candidate gene expression levels. **(A, B)** RT-qPCR validation in an independent cohort. Relative mRNA expression levels of *AHR* **(A)** and *SLC1A5* **(B)** in peripheral blood samples from DR patients (*n* = 5) and healthy controls (*n* = 5). Data are presented as mean ± SD. ** = *p* < 0.01. **(C, D)** In silico validation using the GSE221521 dataset. Expression levels of *AHR* **(C)** and *SLC1A5* **(D)** across three groups: healthy controls (*n* = 50), diabetic patients without retinopathy (DM, n = 74), and DR patients (*n* = 69). Both genes show progressive upregulation from healthy controls to DM and DR.

RT-qPCR validation further showed that both *AHR* and *SLC1A5* were expressed at significantly higher levels in the DR group compared with the control group, which was consistent with the transcriptomic analysis results (**Figures 7A, B**). The primer sequences used for RT-qPCR are listed in **Table 2**.

**Table 2:** List of primer sequences used in the study.

Primer	Gene	Sequence (5′ to 3′)
*AHR-*F	*AHR*	CGTCAGCTACACTGGGCATT
*AHR-*R	*AHR*	ACTACTGTCTGGGGGAGACC
*SLC1A5-*F	*SLC1A5*	AAGTGAAGAGTGAGCTGCCC
*SLC1A5-*R	*SLC1A5*	AGGCATCCCAGATCTCCTGT

To further determine whether the upregulation of *AHR* and *SLC1A5* is specifically associated with DR rather than reflecting diabetes in general, their expression levels were examined in the GSE221521 dataset, which includes healthy controls (*n* = 50), diabetic patients without retinopathy (DM, *n* = 74), and diabetic patients with retinopathy (DR, *n* = 69). Both genes showed a progressive increase in expression across the three groups, with the lowest levels observed in healthy controls, intermediate levels in diabetic patients without retinopathy, and the highest levels in patients with DR (**Figures 7C, D**). These findings suggest that the elevated expression of *AHR* and *SLC1A5* may be more closely associated with DR-related pathological changes rather than representing a general consequence of diabetes.

## 4 Discussion

As a major microvascular complication of hyperglycemia, DR is a primary cause of adult blindness in developed nations (3). Its insidious early progression often delays diagnosis and treatment (47). During the early stages of diabetes, photoreceptor cells in the retina may represent an initial site of abnormal activation of DNL under high-glucose conditions. Excess lipid production in these cells can induce lipotoxicity and lipid spillover, thereby contributing to retinal inflammation and vascular pathology (16). This investigation employed multi-omics data analysis combined with machine learning techniques to pinpoint two DNL-related genes: the aryl hydrocarbon receptor (*AHR*) and solute carrier family 1 member 5 (*SLC1A5*). A nomogram model constructed based on these two candidate genes exhibited preliminary predictive performance for DR risk in research settings. GSEA indicated that these genes participated in several key pathways related to DR pathogenesis. GraphBAN predictions identified two compounds targeting *AHR* and fourteen compounds targeting *SLC1A5*, which were further evaluated using molecular docking and molecular dynamics simulations. scRNA-seq analysis showed that cDC2 and classical monocytes exhibited relatively higher expression of the key genes. Cell communication analysis suggested that cDC2, T and NK cells, pericytes, and non-classical monocytes were major interacting cell populations, with LGALS9−CD45 and ANXA1−FPR1 potentially involved in intercellular communication. *Pseudotime* trajectory analysis showed that cDC2 were divided into two subpopulations and classical monocytes into five. *AHR* displayed similar expression trends during the differentiation of both cell types, whereas *SLC1A5* showed distinct cell-type-specific expression patterns. These findings may provide potential clues for the molecular diagnosis and targeted treatment of DR.

*AHR* encodes an important TF involved in regulating xenobiotic metabolism, immune and inflammatory responses, metabolic homeostasis, and vascular stability. In metabolic regulation, *AHR* participates in DNL-related processes mediated by the tryptophan metabolism axis, and its deficiency has been reported to confer resistance to diet-induced obesity and hepatic steatosis (48, 49). In immune regulation, *AHR* can influence dendritic cell maturation and inflammatory responses by modulating IL-6 and Th17 differentiation (50). In DR, which involves both metabolic dysregulation and chronic inflammation, *AHR* may exert protective effects through multiple mechanisms. On the one hand, *AHR* transcriptionally regulates the expression of the vascular stabilizing factor PEDF, which possesses anti-inflammatory, anti-angiogenic, and neuroprotective properties that help maintain retinal vascular homeostasis (51). On the other hand, through the AHR/IL-17A pathway, it may modulate inflammatory responses; its agonists have been reported to inhibit retinal inflammasome formation, reduce chronic inflammation (52), and improve retinal capillary degeneration (50). These findings suggest that *AHR* may represent a molecular link between metabolic dysregulation and immune-inflammatory responses in DR.

*SLC1A5* encodes a sodium-coupled transporter responsible for the cellular uptake of neutral amino acids, particularly glutamine. Glutamine is critical for maintaining intracellular amino acid balance and serves as a metabolic substrate for multiple biosynthetic pathways, including DNL (53, 54). Glutamine contributes to cellular metabolism in two key ways: providing nitrogen atoms for nucleotide and protein biosynthesis, while simultaneously serving as a principal carbon donor for lipid production. Through glutaminolysis, glutamine is converted to glutamate, which is further metabolized to α-ketoglutarate and enters the tricarboxylic acid cycle. Mitochondrial citrate generated through this process can be transported to the cytosol, where ATP-citrate lyase catalyzes its cleavage to yield acetyl-CoA. This molecule serves as the essential building block for fatty acid biosynthesis (55). This glutamine-dependent lipogenic pathway, also referred to as reductive carboxylation, becomes particularly important under conditions of metabolic stress or limited glucose-derived carbon supply. In this study, *SLC1A5* expression was significantly upregulated in patients with DR. This increase may represent a compensatory response that supports glutamine-dependent metabolism and DNL in retinal cells under hyperglycemic stress. *SLC1A5* has been reported to enhance the resistance of retinal pigment epithelial (RPE) cells to hyperglycemic stress by maintaining glutamine metabolic homeostasis. For example, miR-338-3p can target *SLC1A5* to reprogram metabolism and enhance RPE cell resistance to high-glucose-induced ferroptosis (56). In addition, the deubiquitinating enzyme USP48 stabilizes the *SLC1A5* protein, thereby promoting RPE cell proliferation under high-glucose conditions while inhibiting apoptosis, inflammation, ferroptosis, and oxidative stress (57). The cytoprotective role of *SLC1A5* may also be related to its involvement in supporting DNL. Lipid synthesis is essential for membrane biogenesis, cell proliferation, and the production of lipid signaling molecules. Under diabetic conditions, the demand for these processes may increase, and *SLC1A5*-mediated glutamine uptake may provide carbon precursors to sustain DNL activity. Collectively, these findings demonstrate that *SLC1A5* may serve as a protective regulator in DR pathogenesis, potentially via modulating the metabolism and survival of RPE cells.

RT−qPCR validation in our clinical samples showed that the expression trends of *AHR* and *SLC1A5* were consistent with those in public databases, further confirming the reliability of our analysis. However, a key consideration in interpreting these results is that we used peripheral blood samples, while DR is primarily a local retinal disease, requiring clarification on how blood gene expression reflects local retinal pathology. First, DR, a diabetic complication, does not occur in isolation but with extensive systemic disorders. Key DR drivers (hyperglycemia, oxidative stress, and inflammatory mediators) exert systemic effects on all tissues, including circulating immune cells; thus, peripheral blood gene expression can partially reflect disease-related systemic changes. Second, growing evidence indicates that peripheral immune cells can infiltrate the retina, reflecting and promoting retinal inflammation. Previous studies have confirmed that peripheral blood gene expression profiles can distinguish DR patients from diabetics without retinopathy and correlate with disease severity (58), suggesting an association between blood molecular changes and retinal pathology.

This study further constructed a combined nomogram model incorporating these two genes to evaluate their potential clinical relevance. Through comprehensive validation methods, including calibration curve, DCA, and ROC analysis, the model demonstrated robust predictive performance. These results suggest that the model may hold promise as a candidate decision-support tool for preliminary DR risk stratification in research settings, and indicate that *AHR* and *SLC1A5* may represent potential candidate biomarkers associated with DR.

GSEA showed that the selected genes were significantly enriched in DR-related pathways, including insulin signaling and inflammatory activation. The insulin signaling pathway regulates retinal vascular cell function, neuronal survival, and metabolic balance. Its impairment may lead to pericyte dysfunction, disruption of vascular Angiopoietin1-Tie2 balance, and characteristic DR vascular abnormalities such as retinal venous dilation, increased capillary branching, and abnormal vascular sprouting (59). It may also contribute to retinal neuronal apoptosis through dysfunction of the PI3K-Akt pathway (60). Regarding inflammatory pathways, the TLR signaling pathway may play a role in the inflammatory processes, oxidative stress, and vascular injury observed in DR. TLR and NF-κB signaling are important regulators of immune responses in retinal vascular diseases. Inflammatory responses increase the production of Reactive oxygen species (ROS), leading to oxidative damage and forming a cycle in which inflammation and oxidative stress reinforce each other (61–63). Inhibition of mRNA transcription and protein expression within the TLR signaling pathway has been reported to alleviate inflammatory responses associated with DR (64). Under high-glucose conditions, activation of TLR2/4 can promote the formation of neutrophil extracellular traps (NETs), whose levels are positively correlated with the expression of PADI4, a key enzyme involved in NETs formation (63). Excessive NETs release may lead to vascular endothelial injury, microthrombosis, and amplified inflammatory responses, thereby accelerating DR-related microvascular damage (65). These findings suggest that the identified genes may be associated with DR pathology through their involvement in insulin signaling and inflammatory processes.

The GraphBAN model was used to predict potential gene–compound interactions, identifying two compounds potentially targeting *AHR* and fourteen compounds potentially targeting *SLC1A5*. These compounds may interact with *AHR* or *SLC1A5* and potentially influence related biological processes. Compounds associated with *AHR* may affect inflammatory and oxidative stress pathways mediated by this protein, whereas compounds targeting *SLC1A5* may influence glutamine transport and metabolic homeostasis. In addition, molecular docking and molecular dynamics simulations provided preliminary computer simulation evidence for *AHR* and *SLC1A5* as potential drug targets, suggesting that the screened compounds have a potential binding ability to the target proteins, laying a foundation for future experimental verification and preclinical research.

Single-cell RNA sequencing analysis localized the expression of the identified genes to specific cell populations within DR fibrous membranes, with relatively higher expression observed in cDC2 and classical monocytes. This analysis provides cellular context for the transcriptomic findings by indicating which cell populations contribute to the observed gene expression patterns. cDC2 represents a major subset of myeloid dendritic cells (mDC) and is critically involved in immune defense by activating immune responses against pathogens and tumor cells (66). Previous studies have reported that both the absolute number and proportion of peripheral blood mDC are higher in patients with type 2 diabetes and DR compared with patients with diabetes alone. An imbalance in the mDC/pDC ratio may contribute to Th1/Th2 immune dysregulation and the initiation of adaptive immune-inflammatory responses (67). Classical monocytes, which represent the main subset of circulating monocytes and are often referred to as inflammatory monocytes, are among the first immune cells recruited to sites of infection or tissue injury. Previous studies have shown that monocytes in DR are enriched for genes involved in cytokine signaling and inflammatory activation. Among these, pro-inflammatory CD14++ monocytes appear to play an important role in promoting inflammation by increasing the production of inflammatory cytokines and chemokines. These monocytes, particularly those observed in patients with DME, have been associated with increased activity of inflammatory pathways such as tumor necrosis factor-mediated signaling, the I-κB kinase/NF-κB pathway, and the TLR signaling pathway (68). Given the roles of cDC2 and classical monocytes in antigen presentation and inflammatory responses, interactions between these cell populations may contribute to inflammatory processes in the DR microenvironment. Activated cDC2 may recruit and influence classical monocytes, while inflammatory mediators produced by monocytes may further enhance antigen presentation and T-cell activation by cDC2. Such interactions may contribute to the persistence of inflammatory responses associated with DR.

Cell metabolism analysis showed that these cell populations were enriched in three important metabolic pathways: Glycolysis/Gluconeogenesis, Oxidative Phosphorylation, and Glutathione Metabolism. Glycolysis/Gluconeogenesis is the central bidirectional pathway of glucose metabolism and exhibits significant metabolic reprogramming features in DR, representing an important component of the disease-related metabolic alterations (69). Oxidative Phosphorylation under high-glucose conditions may lead to mitochondrial dysfunction in retinal cells; this abnormality can increase ROS production, thereby inducing oxidative stress and apoptosis and contributing to retinal degenerative changes (70–73). In addition, in the Glutathione Metabolism pathway, glutathione functions as an important antioxidant that helps maintain redox homeostasis. Dysfunction of this pathway may weaken the antioxidant capacity of retinal tissue, aggravate oxidative stress damage, and subsequently promote inflammatory responses and vascular injury (74, 75). These metabolic pathways showed relatively high activity in cDC2 cells. Therefore, targeting cDC2-related metabolic processes may represent a potential strategy for metabolic intervention in DR. Cell communication analysis showed that T cells and NK cells frequently interacted with cDC2, classical monocytes, and pericytes, and exhibited relatively strong communication with classical and non-classical monocytes. Although T cells and NK cells are well documented to be involved in DR, their precise functional roles remain poorly defined (76). As components linking adaptive and innate immunity, they may form inflammatory interaction networks with monocytes and dendritic cells, potentially exacerbating retinal vascular leakage and barrier damage through cytokine release. Their interactions with pericytes may also influence vascular stability. In addition, this study identified two ligand–receptor pairs, LGALS9−CD45 and ANXA1−FPR1, which may participate in communication among classical monocytes and between classical monocytes, cDC2, and non-classical monocytes. *Pseudotim*e analysis showed that *AHR* expression followed a similar pattern in both cDC2 and classical monocytes, with an initial decrease, a slight increase at the intermediate stage, and a subsequent decline. In contrast, *SLC1A5* exhibited cell-type-specific expression patterns. In cDC2, its expression initially decreased, remained relatively stable, and then increased at later stages. In classical monocytes, *SLC1A5* expression showed a slight increase at early stages, followed by a gradual decrease. These findings provide insights into potential cellular state transitions within the DR fibrous membrane microenvironment and highlight dynamic gene expression patterns that may parallel these cellular changes. This analysis provides additional insight into cellular heterogeneity and disease-related processes and may help guide future studies exploring intervention strategies related to these genes. In summary, our exploratory study suggests that *AHR* and *SLC1A5* may represent candidate molecular nodes linking dysregulation of DNL to DR pathogenesis. These genes may contribute to DR-related pathology by influencing metabolic–immune microenvironment homeostasis and participating in pathways such as insulin signaling and TLR signaling. The single-cell analysis indicates that *AHR* and *SLC1A5* are enriched in cDC2 and classical monocytes within DR fibrous membranes, and may be associated with DR-related processes, including retinal vascular inflammation, potentially through regulation of metabolic activity and intercellular communication in these immune cell populations. These preliminary findings provide a foundational reference for further exploring the crosstalk between metabolic and immune mechanisms in DR.

However, several limitations should be considered. First, the sample size in our RT-qPCR validation was relatively small (n = 5 per group), which may increase the risk of statistical bias and limit the generalizability of the results. Therefore, these findings should be considered preliminary and require validation in larger independent cohorts. Second, the experimental design compared DR patients directly with healthy controls without including a diabetic group without retinopathy. This limits our ability to determine whether the observed upregulation of *AHR* and *SLC1A5* is specifically associated with DR pathology or reflects diabetes itself, medication effects, or differences in glycemic control. Third, although basic clinical information was available, our analysis did not fully adjust for important clinical variables such as diabetes duration and medication use. As a result, it remains unclear whether the observed gene expression changes are independently associated with DR severity. Fourth, the analysis was primarily based on transcriptomic and single-cell data obtained from public datasets. Although preliminary validation was performed using RT-qPCR, functional experiments, such as gain-of-function or loss-of-function studies in animal models or larger clinical cohorts, have not yet been conducted. Fifth, the cross-sectional design of this study captures gene expression at a single time point and therefore cannot determine temporal relationships between gene expression changes and disease progression. Finally, the single-cell dataset did not include healthy retinal control samples, which limits the ability to determine whether the cell populations showing higher *AHR* expression are uniquely associated with DR pathology or represent normal cellular components of the fibrous membrane microenvironment. Future studies incorporating larger patient cohorts, functional experiments, and longitudinal designs will be necessary to further clarify the biological roles of these genes and evaluate their potential clinical relevance in DR.

## 5 Conclusion

This exploratory study identified *AHR* and *SLC1A5* as genes associated with DNL-related transcriptional changes in DR and highlighted cDC2 and classical monocytes as cell populations showing relatively higher expression of these genes. In addition, potential compounds targeting these genes were predicted, and their interactions were evaluated through molecular docking and molecular dynamics analyses. These findings provide preliminary insights that may support future mechanistic studies, risk assessment approaches, and exploration of potential therapeutic strategies for DR.

## Data Availability

The original contributions presented in the study are included in the article/**Supplementary Material**. Further inquiries can be directed to the corresponding author.
