# Supplementary material for: Integrative analysis of transcriptomics, single-cell RNA sequencing, and GraphBAN identifies de novo lipogenesis-associated genes and their potential roles in diabetic retinopathy
Source: Front Immunol. 2026 Apr 7;17:1803639. doi: 10.3389/fimmu.2026.1803639 (PMC13095570; doi:10.3389/fimmu.2026.1803639)
Supplement: Supplementary file 1 [file DataSheet1.docx]

Supplementary Material

# Supplementary Figures


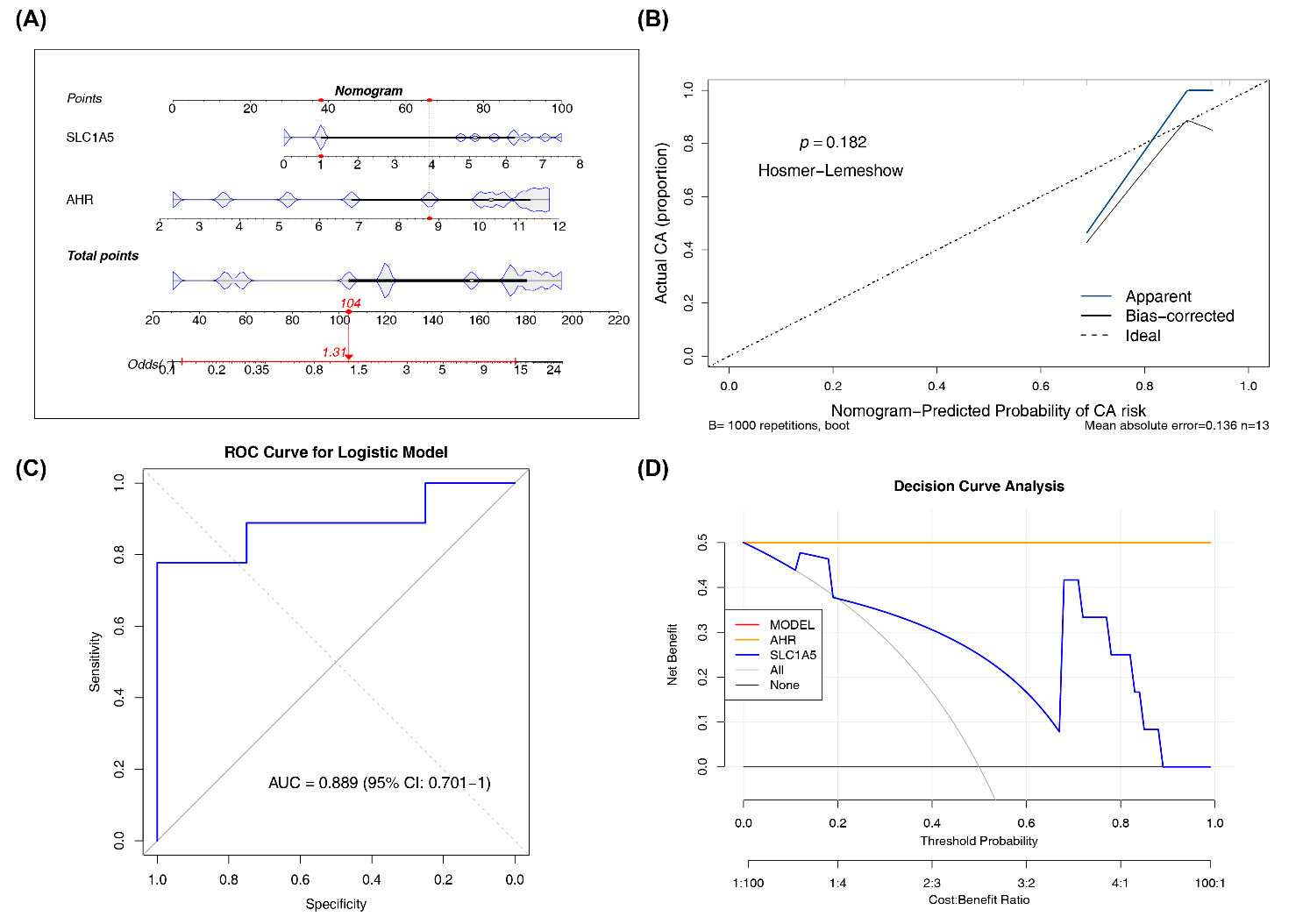


**Supplementary Figure 1. External validation of the nomogram in the independent cohort GSE94019.** (A) The nomogram reconstructed in the validation set GSE94019. (B) Calibration curve of the nomogram in the validation set, showing good agreement between predicted and observed probabilities (HL test, p = 0.182). (C) ROC curve of the nomogram reconstructed in the validation set GSE94019, with an AUC of 0.889, confirming good generalization ability. (D) DCA of the nomogram in the validation set, demonstrating sustained clinical net benefit across a range of threshold probabilities.


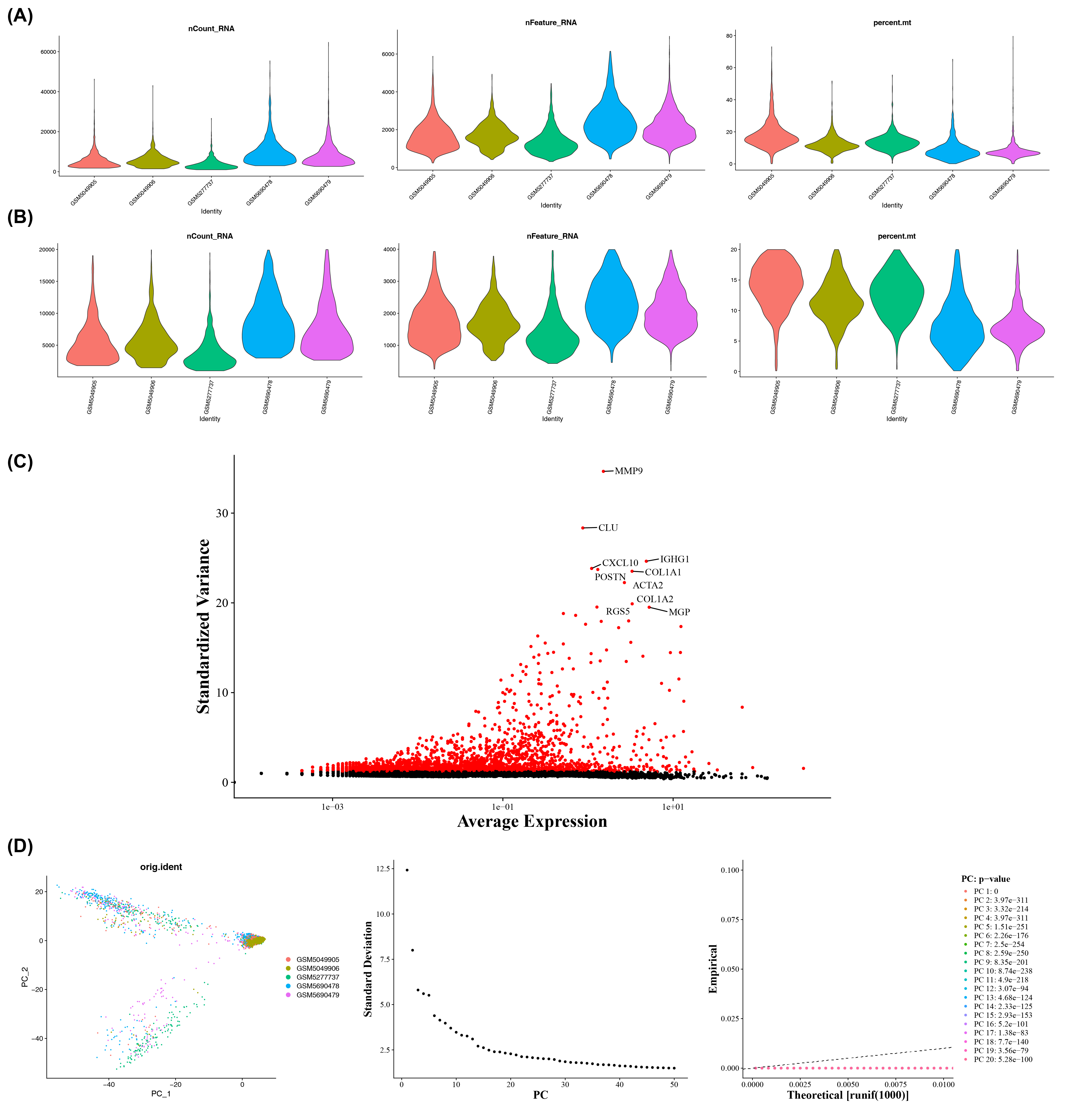


**Supplementary Figure 2.** Quality control, dimensionality reduction, and clustering of single-cell data (A) Before quality control. nFeature_RNA represents the number of expressed genes per cell, nCount_RNA denotes the total gene count per cell, and percent_mt indicates the mitochondrial gene proportion. (B) After quality control. (C) Screening of highly variable genes. The x-axis represents the average gene expression level, and the y-axis represents the normalized variance of gene expression; red dots indicate highly variable genes. (D) Principal component analysis (PCA) plot of sample cells. The statistical significance of each principal component (PC) was evaluated by comparing the empirical distribution of PCs with the theoretical uniform distribution. The standard deviation of PCA tended to stabilize after 20 PCs.

1. **Supplementary Tables**

**Supplementary Table 1.** The 575 DNL-related genes.

**Supplementary Table 2.** GO and KEGG enrichment analyses.

**Supplementary Table 3.** Validation of Adjusted P-values for Key Genes

**Supplementary Table 4.** GSEA of key genes.

**Supplementary Table 5.** Clinical Characteristics of RT-qPCR Samples.
