# Supplementary material for: Integrative analysis of transcriptomics, single-cell RNA sequencing, and GraphBAN identifies de novo lipogenesis-associated genes and their potential roles in diabetic retinopathy
Source: Front Immunol. 2026 Apr 7;17:1803639. doi: 10.3389/fimmu.2026.1803639 (PMC13095570; doi:10.3389/fimmu.2026.1803639)
Supplement: Supplementary file 4 [file Table3.docx]

Supplementary Table 3. Validation of Adjusted P-values for Key Genes

|  | log2FoldChange | pvalue | padj |
| --- | --- | --- | --- |
| AHR | 0.784113074968503 | 1.17229335362488e^-11^ | 9.8927912578307e^-09^ |
| SLC1A5 | 0.551908158425868 | 4.02379587996608e^-07^ | 1.5541911586369e^-05^ |
